# BiComp-DTA: Drug-target binding affinity prediction through complementary biological-related and compression-based featurization approach

**DOI:** 10.1371/journal.pcbi.1011036

**Published:** 2023-03-31

**Authors:** Mahmood Kalemati, Mojtaba Zamani Emani, Somayyeh Koohi

**Affiliations:** Department of Computer Engineering, Sharif University of Technology, Tehran, Iran; Icahn School of Medicine at Mount Sinai, UNITED STATES

## Abstract

Drug-target binding affinity prediction plays a key role in the early stage of drug discovery. Numerous experimental and data-driven approaches have been developed for predicting drug-target binding affinity. However, experimental methods highly rely on the limited structural-related information from drug-target pairs, domain knowledge, and time-consuming assays. On the other hand, learning-based methods have shown an acceptable prediction performance. However, most of them utilize several simple and complex types of proteins and drug compounds data, ranging from the protein sequences to the topology of a graph representation of drug compounds, employing multiple deep neural networks for encoding and feature extraction, and so, leads to the computational overheads. In this study, we propose a unified measure for protein sequence encoding, named BiComp, which provides compression-based and evolutionary-related features from the protein sequences. Specifically, we employ Normalized Compression Distance and Smith-Waterman measures for capturing complementary information from the algorithmic information theory and biological domains, respectively. We utilize the proposed measure to encode the input proteins feeding a new deep neural network-based method for drug-target binding affinity prediction, named BiComp-DTA. BiComp-DTA is evaluated utilizing four benchmark datasets for drug-target binding affinity prediction. Compared to the state-of-the-art methods, which employ complex models for protein encoding and feature extraction, BiComp-DTA provides superior efficiency in terms of accuracy, runtime, and the number of trainable parameters. The latter achievement facilitates execution of BiComp-DTA on a normal desktop computer in a fast fashion. As a comparative study, we evaluate BiComp’s efficiency against its components for drug-target binding affinity prediction. The results have shown superior accuracy of BiComp due to the orthogonality and complementary nature of Smith-Waterman and Normalized Compression Distance measures for protein sequences. Such a protein sequence encoding provides efficient representation with no need for multiple sources of information, deep domain knowledge, and complex neural networks.

## Author summary

Drugs alter the protein conformation and functionality through binding to the target proteins. Measuring the binding tendency between drugs and proteins, known as drug-target binding affinity prediction, is an important task in developing new drug candidates and novel therapeutics in the early stage of drug discovery. Measuring the affinity strength through biological assays is expensive and time-consuming. On the other hand, considerable data-driven and computational methods are proposed to predict binding affinity prediction through employing complicated models, and multiple types of proteins and drug compounds data. In this paper, we present a deep learning-based method, named BiComp-DTA, which utilizes both biological and sequence content features extracted from the protein sequences using a new unified measure, named BiComp. Encoded protein sequences and drug’s information are passed to the fully-connected and hybrid neural networks, including CNN and separable CNN layers, respectively. Simulation results using benchmark datasets indicate that BiComp-DTA provides an accurate and fast prediction for drug-target binding affinity prediction without employing multiple complicated networks, several types of proteins and drug compounds data, and external tools for input data modeling.

## 1. Introduction

Prediction of the interaction strength between biomolecules (i.e. proteins or targets) and their binding partners (i.e. ligands or compounds) is a crucial early step in drug discovery and drug repurposing processes [1]. Traditionally, determination of the binding affinity between candidate ligands and protein targets are accomplished by the expensive and time-consuming wet-lab experiments. There are various approaches to experimentally determine ligand-binding affinities including calorimetric methods, such as isothermal titration calorimetry (ITC) [2], optimal spectroscopy methods, particularly fluorescence anisotropy/polarization-based assays (FA/FP) [3], surface plasmon resonance (SPR) approaches [4], and immunoprecipitation–based approaches, such as quantitative western blotting [5]. Furthermore, there are high-throughput screening methodologies, such as microarrays [6,7], but careful analysis of binding affinities for individual protein-ligand pairs is generally performed via other experimental approaches.

Hence, several cost-effective and fast computational methods have been proposed so far to predict drug-target binding affinity, among which, machine learning-based computational methods have shown the capability of streamline and effective drug-target binding affinity prediction [8–12].

Traditional machine learning-based computational methods formulate the drug-target interaction prediction as a binary classification problem, where the model predicts whether a drug-target pair could be bound. Recently, various learning-based methods have been proposed to predict the continuous binding affinity values by framing the problem as a regression task [8–12]. These methods aimed to improve the prediction output by adopting various feature selection and feature extraction approaches to efficiently represent the input data based on the available drug and target data. The selected or extracted features are fed to either a traditional machine learning-based model or a deep neural network to perform the prediction task. Therefore, efficient construction of the input features, as well as the network architecture learning the representations, play a key role in the prediction model.

Earlier machine learning-based methods for drug-target binding affinity prediction utilize the similarity-based features from several sources of data. KronRLS [8] incorporates the similarity of the drug’s chemical structure, as well as the Smith-Waterman [13] similarity score of target sequences, to feed the prediction model, built upon the Kronecker Regularized Least Squares algorithm. Despite its ability to predict continuous affinity values, the method cannot consider the non-linear complex associations [9] due to the adopted linear algorithm. To consider the interaction features between the drug and target sequences, SimBoost [9] utilizes their similarities and network-based interaction features for feeding a prediction model based on the gradient boosting machine. Although the method utilizes several sources of information, the constructed features for proteins and drugs rely on limited evolutionary-based and 2D representation information, respectively. To extract deep features from similarity information, SimCNN-DTA adopted 2D-CNN layers for feature extraction from the outer product of the drug and target similarity matrices [10]. Despite employing a nonlinear deep learning-based architecture, the method relies on the representation of the inputs based on the drugs and proteins’ similarities, and so, it cannot extract enough informative features. On the other hand, the method proposed in [11], as well as NerLTR-DTA [12] are developed based on the gradient boosting models utilizing multiple sources of information. Taking advantage of multiple sources of information, the aforementioned methods suffer from resource-extensive preprocessing and time-consuming information extraction. Furthermore, the prediction models built upon the machine learning algorithms may overemphasize outliers and cause overfitting [14].

To overcome several aforementioned disadvantages of traditional similarity-based methods, deep learning-based DTA methods have been proposed to enable highly informative feature extraction from both protein and drug sequences. DeepDTA [15], as an earlier deep learning-based method, proposes a CNN-based model for feature extraction from the protein sequences and the SMILES format [16] of the drug sequences. Furthermore, this method utilizes Smith-Waterman and CNN-based features for protein and drug encoding, respectively. Despite automatic feature extraction, the model has been trained on the limited available labeled sequence data. To employ richer data sources, WideDTA [17] incorporated two extra types of sequence information, including protein domains and motifs, and ligand maximum common substructures. It should be noted that the prediction accuracy comes at the cost of several input data and CNN blocks, which in turn increases the network’s complexity. For more informative feature extraction, AttentionDTA [18], and MATT-DTI [19] adopt extra attention layers alongside the CNN blocks. Specifically, MATT-DTI utilizes multiple attention blocks, including the relative self-attention and the multi-head attention layers for drug and interaction representation, respectively. Moreover, in recent studies [20,21], mutual and multilevel attention mechanisms are adopted to capture the drug-target pair attention scores along with the 1D-CNN and very deep 2D-CNN blocks, respectively. Although these attention-based methods accomplish an automatic feature extraction solely from the sequence data, the training procedure depending on the available labeled data may not be sufficient for learning the distributed representation of proteins and drugs. Finally, extra attention mechanisms improve prediction accuracy at the cost of increased network complexity.

To overcome the scarcity of labeled data for extracting distributed representation, transformer-based methods have been proposed for DTA prediction, thanks to the transformer architectures [22] proposed for natural language processing (NLP) tasks. MT-DTI [23] incorporates a molecular transformer along with the CNN blocks for drug and protein sequence encoding, respectively. Despite generating informative distributed representation vectors, the network architecture suffers from the overall complexity. Recently, FusionDTA [24] adopted ESM-1b [25] transformer for generating distribution representation vectors from the protein sequences. Although this method has shown promising performance, it relies on extra pre-training and fine-tuning stages for efficient protein sequence encoding. In general, the transformer-based methods require excessive memory requirements and rely on many-core GPU or TPU machines for training the model [23,26]. Furthermore, since the learning representation of the input data involves limited sequence information, the informative structural information cannot be considered for the feature extraction task.

To improve feature extraction, graph neural network-based methods have been proposed, which utilize structural information of the drugs and targets. For example, GraphDTA [27] utilizes various atomic features for drug compounds and employs four GNNs to learn the drug’s graph representation. However, this method does not consider graph representation for the protein sequences. To resolve this issue, DGraphDTA [28] utilizes GNNs for learning the graphs representation from both the constructed drug molecular graphs and the protein graphs. The latter capability improves the prediction performance at the cost of involving extra toolkits and methods for predicting the contact maps and constructing the graph models. To capture local and global structures of the drug’s graph-based representation in a parallel manner, MGraphDTA [29] has been proposed, which incorporates very deep GNNs, and three branches of CNN blocks for representing the drug’s graph and the protein sequence, respectively, which in turn leads to the computational overheads in terms of time and space complexity. For capturing structural, spatial, and sequential features, DeepH-DTA [30] proposes a model based on the heterogeneous graph attention (HGAT) [31] and bidirectional ConvLSTMs [32] for the drugs sequences along with the squeezed-excited dense CNN blocks for the protein sequences. In this manner, this method suffers from the time and space complexity of the model. Besides the aforementioned drawbacks of the GNN-based methods, generalizing GNNs to the larger and noisy graphs may necessitate utilization of extra attention mechanisms, which in turn increases computational overheads in terms of time and memory usage [33,34]. Furthermore, local dependency consideration by the GNN models cannot learn global features of the graphs, including recurring patterns and motifs, efficiently [34,35].

In all, various feature extraction approaches have shown promising performance for drug-target binding affinity prediction. Generally, similarity-based methods rely on expert knowledge, as well as several preprocessing steps for constructing multiple input matrices feeding a ML-based model. On the other hand, sequence-based deep learning methods extract features from a limited set of labeled sequence data, which cannot provide highly informative input’s representation for the DTA prediction. Meanwhile, transformer-based methods suffer from the time and space complexity overheads for generating distributed representation vectors. Finally, GNN-based methods, besides depending on the external toolkits and methods for graph generation, rely on very deep models for capturing informative local and global features. To overcome the aforementioned challenges, fast and cost-effective computation, in addition to high prediction accuracy, should be addressed in a DTA prediction approach.

For this purpose, in this paper, we propose a deep learning-based method, named BiComp-DTA, for drug-target binding affinity predictions. For capturing informative features from the protein sequences, we propose a unified measure constructed upon an alignment-free (i.e. Lempel–Ziv–Markov chain algorithm (LZMA)) [36] and an alignment-based (i.e. Smith-Waterman) similarity measures, named BiComp, for DTA prediction. It should be noted that the utility of alignment-based and alignment-free-based distance measures and their complementary nature for protein sequence comparison and classification has been shown in [37,38], by constructing the Lempel-Ziv-Welch (LZW)-BLAST measure using the combining LZW and BLAST scores [38]. On the other hand, the utility of similarity-based information, such as Smith-Waterman scores, has been shown in the DTA prediction task [9,10,15], but their prediction performance is poor, or depends on employing multiple information types. Motivated by these works, we proposed the hypothesis of employing LZMA alongside with Smith-Waterman to construct the unified measure for the DTA prediction task.

Moreover, adopting a fully-connected network for information extraction from the protein features provides a fast and accurate representation of protein sequences, with no need to complex neural network architectures. For an accurate and cost-effective feature extraction from the drug sequences in SMILES format, we propose the adoption of CNNs along with the separable convolution layer. In all, our method includes four major parts: a) protein sequences encoding using the unified measure (i.e. BiComp), b) a fully-connected neural network for feature extraction from the encoded protein sequences, c) a separable CNN layer along with two CNN layers for feature extraction from the drug sequences in SMILES format, and d) a fully-connected neural network for predicting continuous binding affinity values from the concatenated representations of proteins and drugs. In all, the main contributions of the BiComp-DTA can be summarized as follows:

- For efficient protein representation, we propose a unified measure based on the evolutionary-related and the compression-based scores, extracted from an alignment-based (i.e. Smith-Waterman) and an alignment-free (i.e. LZMA) algorithms, respectively. In this manner, the unified measure provides a complementary feature based on the algorithmic information theory, as well as preserves the evolutionary relationship between various protein sequences for the DTA prediction.
- We propose a deep neural network architecture adopting CNN blocks followed by a separable convolution layer for learning an informative drug representation from the drug in SMILES format. The network can provide an accurate representation while preventing computational overheads, in terms of the number of trainable parameters.
- BiComp-DTA provides an efficient DTA prediction architecture neither utilizing complex and very deep neural networks nor constructing and employing multiple sources of divergent information.

## 2. Results

We compared the performance of BiComp-DTA for drug-target binding affinity prediction with some of the state of the art methods, using four metrics, Concordance Index (CI), Mean Squarer Error (MSE), a widely-used metric, proposed in [39] to validate the external prediction performance of a quantitative structure-activity relationship (QSAR) model ($r_{m}^{2}$), and Area Under Precision Recall (AUPR), as mentioned in S1 Text. In this study, two widely-used datasets, known as Davis [40] and Kiba [41] datasets, and two recently updated datasets, known as BindingDB [42] and PDBbind [43] datasets, have been considered as the benchmark datasets, which are explored as follows. The Davis dataset contains the binding affinity values of kinase inhibitors with kinases covering near 80% of the human catalytic protein kinome [42]. The Kiba dataset contains drug-target binding affinity information captured by the various bioactivity types, including the inhibition constant (Ki), the dissociation constant (Kd), and the half maximal inhibitory concentration (IC50) [41,42]. The BindingDB dataset contains measured drug-target binding affinities from various sources, including patents, journals, and assays [42]. The PDBbind dataset provides experimentally measured binding affinity information for various protein-ligand complexes stored in the Protein Data Bank (PDB) [43]. More detailed information of the benchmark datasets have been provided in Section Material and Methods.

Furthermore, we evaluated and compared the utility of BiComp-DTA for DTA prediction for unseen data by applying multiple alternative data splitting settings. Moreover, we conducted various adversarial control experiments on straw models to ensure that our models do not fit confounding variables and data artifacts. Furthermore, we conducted feature ablation experiments to explore the ability of BiComp measure to provide an accurate DTA prediction as well as to investigate the contribution of the encoding components. Moreover, we evaluated the performance of BiComp-DTA against multiple simple baseline models to contextualize the predictive power of the models.

Moreover, we compared the network complexity of BiComp-DTA with that of the alternative methods, in terms of the number of trainable parameters. Finally, runtime of the BiComp-DTA is compared against that of the existing methods on GPUs, as well as a normal desktop system. The implementation and performance evaluation details are provided in S1 Text.

For the aforementioned comparative studies, we categorized existing methods into four groups based on the employed data encoding and the feature extraction methods.

- Similarity-based DTA methods. KronRLS, SimBoost, Sim-CNN-DTA, [11], and NerLTR-DTA extract various similarity-based features from drugs and targets. Sim-CNN-DTA employs CNNs for feature extraction from the constructed input data, while the others employ a machine learning based algorithm.
- Sequence-based DTA methods. DeepDTA, WideDTA, AttentionDTA, [20,21], and MATT-DTI utilize the input data sequences encoded by a simple label-encoding method. They employ CNNs either with or without attention mechanisms for feature extraction from the protein sequences and drugs in the SMILES format.
- Transformer-based DTA methods. MT-DTI and FusionDTA incorporate transformers for drug sequences in the SMILES format and protein sequences, respectively.
- Graph neural network-based DTA methods. GraphDTA, DGraphDTA, MGraphDTA, and DeepH-DTA employ GNNs for learning either the drug or protein representations.

### 2.1 Comparing BiComp-DTA to baselines in terms of the accuracy metrics

Tables 1 and 2 compare performance of the BiComp-DTA against the alternative methods in terms of CI, MSE, $r_{m}^{2}$ and AUPR for two widely-use benchmark datasets, Davis and Kiba.

**Table 1:** The CI, MSE, $r_{m}^{2}$ and AUPR values for the BiComp-DTA compared to the alternative methods–Davis dataset.

	Proteins	Drugs	CI(std)	MSE	$r_{m}^{2}$(std)	AUPR(std)
KronRLS	SW	PubChem	0.871(0.001)	0.379	0.407 (0.005)	0.661 (0.010)
SimBoost	SW	PubChem	0.872 (0.002)	0.282	0.644 (0.006)	0.709 (0.008)
SimCNN-DTA	SW-2DCNN	PubChem-2DCNN	0.852 (0.002)	0.319	0.595 (0.01)	0.657 (0.007)
DeepDTA	L.E+ E.L+ 1DCNN	L.E+ E.L+ 1DCNN	0.878 (0.004)	0.261	0.630 (0.017)	0.714 (0.010)
	SW	L.E+ E.L+ 1DCNN	0.886 (0.008)	0.420	0.557 (0.047)	0.713 (0.028)
WideDTA	L.E+ E.L+ 1DCNN L.E+ E.L+ 1DCNN	L.E+ E.L+ 1DCNN L.E+ E.L+ 1DCNN	0.886 (0.003)	0.262	-	-
AttentionDTA	L.E+ E.L+ 1DCNN	L.E+ E.L+ 1DCNN	0.893 (0.005)	0.216	0.677 (0.024)	0.776 (0.024)
MATT-DTI	L.E+ E.L + 1DCNN	L.E+ E.L+SA+FC+ 1DCNN	0.891 (0.003)	0.227	0.683 (0.009)	-
GraphDTA	L.E+ E.L+ 1DCNN	GNN	0.884 (0.002)	0.258	0.656 (0.014)	0.710 (0.006)
FusionDTA	Transformer + FF+ BiLSTM	FF + BiLSTM	0.903 (0.002)	0.220	0.666 (0.008)	0.773 (0.008)
BiComp-DTA	BiComp +FC	L.E+ E.L+ 1DCNN+SepCNN	0.904 (0.001)	0.237	0.696 (0.012)	0.753 (0.006)

SW, PubChem, L.E, E.L, SA, FC, 1DCNN, 2DCNN, SepCNN, GNN, FF, and BiLSTM stand for Smith-Waterman similarity, PubChem similarity, label encoding, embedding layer, Self-Attention block, fully-connected block, one-dimensional convolutional neural networks, two-dimensional convolutional neural networks, separable CNN, Graph neural networks, Feedforward layer, and bidirectional LSTM block, respectively.

**Table 2:** The CI, MSE, $r_{m}^{2}$ and AUPR values for the BiComp-DTA compared to the alternative methods—Kiba dataset.

	Proteins	Drugs	CI(std)	MSE	$r_{m}^{2}$(std)	AUPR(std)
KronRLS	SW	PubChem	0.782 (0.001)	0.411	0.342 (0.001)	0.635 (0.004)
SimBoost	SW	PubChem	0.836 (0.001)	0.222	0.629 (0.007)	0.760 (0.003)
SimCNN-DTA	SW-2DCNN	PubChem-2DCNN	0.821 (0.001)	0.274	0.573 (0.003)	0.721 (0.001)
DeepDTA	L.E+ E.L+ 1DCNN	L.E+ E.L+ 1DCNN	0.863 (0.002)	0.194	0.673 (0.009)	0.788 (0.004)
	SW	L.E+ E.L+ 1DCNN	0.854 (0.001)	0.204	0.692 (0.009)	0.786 (0.005)
WideDTA	L.E+ E.L+ 1DCNN L.E+ E.L+ 1DCNN	L.E+ E.L+ 1DCNN L.E+ E.L+ 1DCNN	0.875 (0.001)	0.179	-	-
AttentionDTA	L.E+ E.L+ 1DCNN	L.E+ E.L+ 1DCNN	0.882 (0.004)	0.155	0.755 (0.017)	0.829 (0.005)
MATT-DTI	L.E+ E.L+ 1DCNN	L.E+ E.L+SA+FC+ 1DCNN	0.889 (0.001)	0.150	0.756 (0.011)	-
GraphDTA	L.E+ E.L+ 1DCNN	GNN	0.879 (0.004)	0.162	0.736 (0.028)	0.823 (0.009)
FusionDTA	Transformers + FF+ BiLSTM	FF + BiLSTM	0.890 (0.001)	0.167	0.699 (0.010)	0.831 (0.003)
BiComp-DTA	BiComp +FC	L.E+ E.L+ 1DCNN+SepCNN	0.891(<0.001)	0.167	0.757 (0.012)	0.834 (0.003)

SW, PubChem, L.E, E.L, SA, FC, 1DCNN, 2DCNN, SepCNN, GNN, FF, and BiLSTM stand for Smith-Waterman similarity, PubChem similarity, label encoding, embedding layer, Self-Attention block, fully-connected block, one-dimensional convolutional neural networks, two-dimensional convolutional neural networks, separable CNN, Graph neural networks, Feedforward layer, and bidirectional LSTM block, respectively.

According to Tables 1 and 2, BiComp-DTA outperformed all baseline methods for DTA prediction in both datasets in term of the first and third accuracy metrics, CI and $r_{m}^{2}$. Furthermore, BiComp-DTA outperformed all methods in terms of the fourth metric, AUPR, for Kiba dataset with comparable MSE for both datasets. Taking advantages of the new proposed measure for protein sequence encoding, applying a fully connected network for feature extraction from the protein sequences, and finally, applying a CNN block including a separable CNN layer, BiComp-DTA outperformed the alternative DTA methods.

To examine the performance of BiComp-DTA in more details, we considered two recently published benchmark datasets, BindingDB and PDBBind datasets, as well. To this end, we evaluated and compared BiComp-DTA against some alternative methods, as reported in Tables 3 and 4. These tables compare performance of the BiComp-DTA, examining BindingDB and PDBbind datasets, against two versions of DeepDTA, one utilizes SW similarity scores (i.e. DeepDTA-Sim) while the other one takes advantages of CNN-based (i.e. DeepDTA-CNN) features for protein sequences, GraphDTA as a graph-based method, and FusionDTA as a transformer-based method.

**Table 3:** The CI, MSE, $r_{m}^{2}$ and AUPR values for the BiComp-DTA compared to the alternative methods—BindingDB dataset.

	Proteins	Drugs	CI(std)	MSE	$r_{m}^{2}$(std)	AUPR(std)
DeepDTA-Sim	SW	L.E+ E.L+ 1DCNN	0.810 (0.007)	0.726	0.555 (0.053)	0.753 (0.024)
DeepDTA-CNN	L.E+ E.L+ 1DCNN	L.E+ E.L+ 1DCNN	0.838 (0.003)	0.558	0.589 (0.015)	0.806 (0.007)
GraphDTA	L.E+ E.L+ 1DCNN	GNN	0.858 (0.001)	0.427	0.712 (0.019)	0.858 (0.006)
FusionDTA	Transformers + FF+ BiLSTM	FF + BiLSTM	0.863 (0.001)	0.466	0.652 (0.010)	0.840 (0.007)
BiComp-DTA	BiComp +FC	L.E+ E.L+ 1DCNN+SepCNN	0.866 (0.001)	0.443	0.671 (0.013)	0.860 (0.007)

SW, L.E, E.L, FC, 1DCNN, SepCNN, GNN, FF, and BiLSTM stand for Smith-Waterman similarity, label encoding, embedding layer, fully-connected block, one-dimensional convolutional neural networks, separable CNN, Graph neural networks, Feedforward layer, and bidirectional LSTM block, respectively.

**Table 4:** The CI, MSE, $r_{m}^{2}$ and AUPR values for the BiComp-DTA compared to the alternative methods—PDBbind dataset.

	Proteins	Drugs	CI(std)	MSE	$r_{m}^{2}$(std)	AUPR(std)
DeepDTA-Sim	SW	L.E+ E.L+ 1DCNN	0.745 (0.004)	2.219	0.408 (0.011)	0.748 (0.006)
DeepDTA-CNN	L.E+ E.L+ 1DCNN	L.E+ E.L+ 1DCNN	0.756 (0.005)	2.140	0.471 (0.025)	0.777 (0.017)
GraphDTA	L.E+ E.L+ 1DCNN	GNN	0.747 (0.005)	1.999	0.402 (0.010)	0.747 (0.011)
FusionDTA	Transformers + FF+ BiLSTM	FF+ BiLSTM	0.768 (0.003)	1.936	0.462 (0.017)	0.782 (0.009)
BiComp-DTA	BiComp +FC	L.E+ E.L+ 1DCNN+SepCNN	0.776 (0.003)	1.983	0.496 (0.022)	0.783 (0.012)

SW, L.E, E.L, FC, 1DCNN, SepCNN, GNN, FF, and BiLSTM stand for Smith-Waterman similarity, label encoding, embedding layer, fully-connected block, one-dimensional convolutional neural networks, separable CNN, Graph neural networks, Feedforward layer, and bidirectional LSTM block, respectively.

According to Tables 3 and 4, in terms of CI and AUPR, BiComp-DTA outperformed all baseline methods for DTA prediction in both datasets, while it provided the second best MSE for BindingDB and PDBbind datasets, respectively. Furthermore, BiComp-DTA provided best $r_{m}^{2}$ compared to the baseline methods for PDBbind dataset, while it provided the second best $r_{m}^{2}$ for BindingDB dataset, respectively.

**Fig 1:**
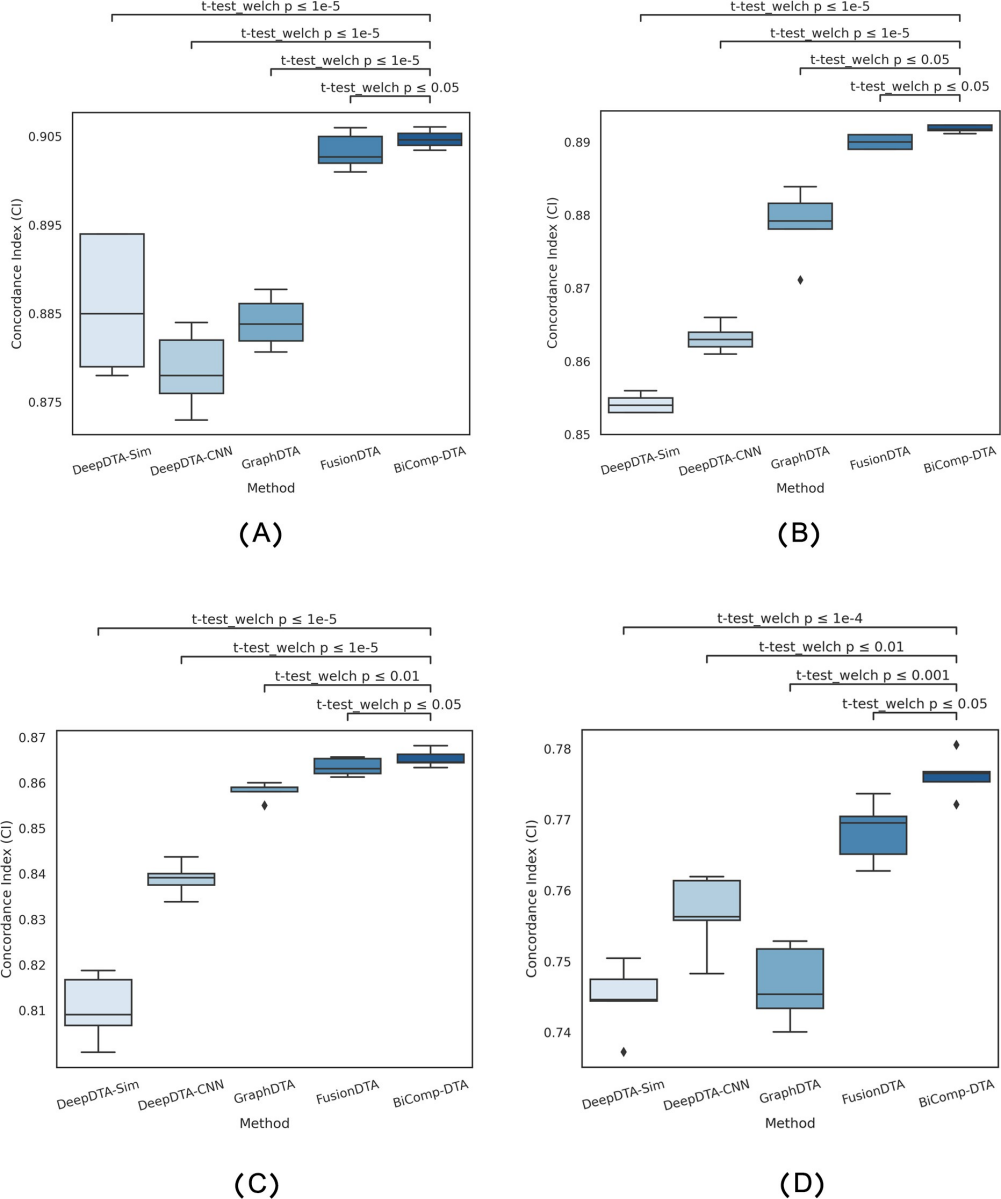
Full distribution of CI values and statistical test results for the BiComp-DTA, compared to the alternative methods for (A) Davis, (B) Kiba, (C) BindingDB, and (D) PDBbind datasets.

To demonstrate that the performance improvements of BiComp-DTA, as compared to baselines, are statistically significant for all datasets, we conducted various statistical tests for all aforementioned experiments. Specifically, we employed Welch’s t-test due to the unequal variances between groups, and apply Bonferroni correction for multiple testing through our analysis. We considered the null hypothesis that mean CI gain for BiComp-DTA equals zero. Fig 1 represents the distribution of CI values for DeepDTA-Sim, DeepDTA-CNN, GraphDTA, FusionDTA, and BiComp-DTA. The relevant adjusted p-values for BiComp-DTA and alternative methods on CI are annotated on the plot. According to Fig 1, the results of statistical tests suggest that BiComp-DTA improved performance prediction by more than 95% significance for all datasets.

### 2.2 Performance comparison of the predicted and actual binding values

**Fig 2:**
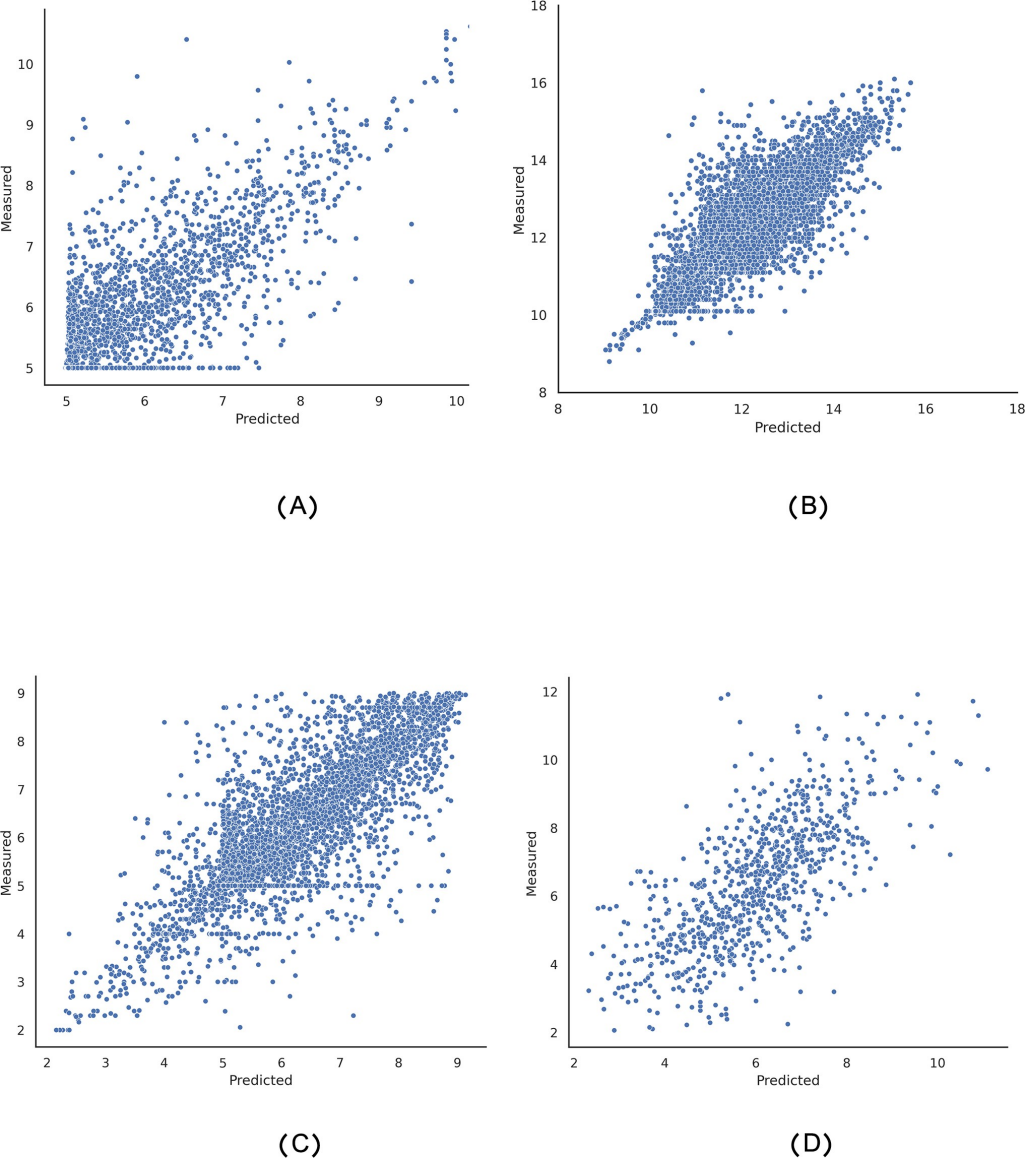
Predicted and actual binding affinities for the (A) Davis, (B) Kiba, (C) BindingDB, and (D) PDBbind datasets.

In this section, we compared the predicted and actual binding values for the four benchmark datasets. For this purpose, we measured the closeness of actual and predicted binding affinity values. As shown in Fig 2, the predicted affinity values, confirm that BiComp-DTA predicts drug-target binding affinities very close to the ground-truth values, for all Davis, Kiba, BindingDB, and PDBbind datasets.

### 2.3 Comparing BiComp-DTA to baselines in terms of the accuracy metrics for non-redundant data setting evaluations

Redundancy and biases in the data, as the result of multiple annotations for similar proteins and similar ligands, can affect performance of the DTA prediction methods. To address this issue, we evaluated and compared the proposed method against alternative methods on a refined version of the Davis dataset. For this purpose, we excluded the redundant protein sequences (i.e. almost 15% of the total protein sequences) from the Davis dataset. In this regard, we trained and evaluated the models with a smaller version of the dataset, and so, without redundant protein sequences. Table 5 provides the comparison of BiComp-DTA, against alternative methods for the refined Davis dataset.

**Table 5:** The CI, MSE, $r_{m}^{2}$ and AUPR values for the BiComp-DTA compared to the alternative methods—refined Davis dataset.

	Proteins	Drugs	CI(std)	MSE	$r_{m}^{2}$(std)	AUPR(std)
DeepDTA-Sim	SW	L.E+ E.L+ 1DCNN	0.871(0.003)	0.574	0.592 (0.041)	0.696 (0.016)
DeepDTA-CNN	L.E+ E.L+ 1DCNN	L.E+ E.L+ 1DCNN	0.869 (0.004)	0.259	0.616 (0.034)	0.695 (0.027)
GraphDTA	L.E+ E.L+ 1DCNN	GNN	0.875 (0.001)	0.226	0.651 (0.014)	0.717 (0.017)
FusionDTA	Transformers + FF+ BiLSTM	FF+ BiLSTM	0.898 (0.002)	0.194	0.664 (0.011)	0.755 (0.011)
BiComp-DTA	BiComp +FC	L.E+ E.L+ 1DCNN+SepCNN	0.901 (0.001)	0.212	0.666 (0.007)	0.746 (0.010)

SW, L.E, E.L, FC, 1DCNN, SepCNN, GNN, FF, and BiLSTM stand for Smith-Waterman similarity, label encoding, embedding layer, fully-connected block, one-dimensional convolutional neural networks, separable CNN, Graph neural networks, Feedforward layer, and bidirectional LSTM block, respectively.

According to Tables 5, in terms of CI and $r_{m}^{2}$, BiComp-DTA outperformed all baseline methods for DTA prediction, while it provided the second best MSE and AUPR in the refined Davis dataset, respectively.

**Fig 3:**
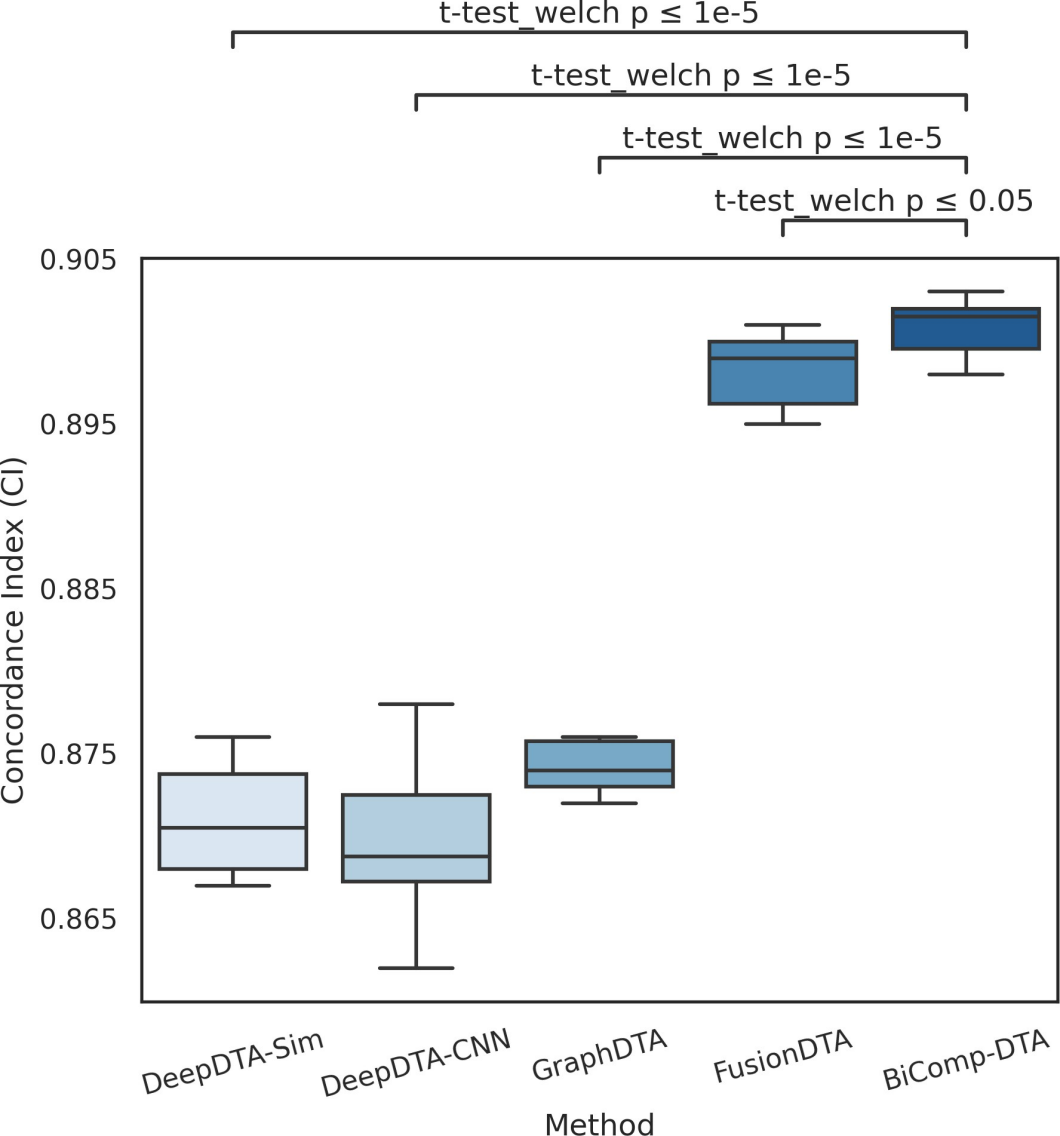
Full distributions of CI values and statistical tests for the BiComp-DTA, compared to the alternative methods for the refined Davis dataset.

Fig 3 represents the distribution of CI values for DeepDTA-Sim, DeepDTA-CNN, GraphDTA, FusionDTA, and BiComp-DTA for the refined Davis dataset experiment. The relevant adjusted p-values with Bonferroni corrections for BiComp-DTA, against alternative methods, on CI are annotated on the plot. According to this figure, the statistical test results suggest that BiComp-DTA improved performance prediction with above 95% significance for the clean version of Davis (i.e. refined Davis) dataset experiment. Hence, BiComp-DTA can be utilized for DTA prediction for the non-redundant data setting.

### 2.4 Comparing BiComp-DTA to baselines in terms of the accuracy metrics for cold-start settings evaluations

To evaluate the method’s robustness and generalization, especially for unseen data, we conducted widely-used alternative splitting data settings, named cold-start settings [24,44]. For this purpose, three settings have been applied for training and testing the method, including cold-protein, cold-drug, and cold-drug-protein for which, the model testing is performed for unseen protein, unseen drug, and unseen drug-protein pairs in the training set, respectively. Hence, these settings are more challenging than the warm-setting problem, for which all the drugs and proteins in the test set can also exist in the training set. Table 6 provides the comparison results for BiComp-DTA, against the alternative methods, considering the cold-start settings for the Davis dataset.

**Table 6:** The CI and MSE values for the BiComp-DTA compared to the alternative methods considering cold-start settings- Davis dataset.

	Proteins	Drugs	Cold-Protein		Cold-Drug		Cold-Protein-Drug	
			CI(std)	MSE	CI(std)	MSE	CI(std)	MSE
DeepDTA-Sim	SW	L.E+ E.L+ 1DCNN	0.846 (0.006)	1.072	0.635 (0.055)	0.569	0.734 (0.021)	0.994
DeepDTA-CNN	L.E+ E.L+ 1DCNN	L.E+ E.L+ 1DCNN	0.810 (0.011)	0.444	0.602 (0.020)	0.671	0.690 (0.038)	0.740
GraphDTA	L.E+ E.L+ 1DCNN	GNN	0.778 (0.005)	0.502	0.720 (0.042)	0.416	0.685 (0.029)	0.757
FusionDTA	Transformers + FF+ BiLSTM	FF + BiLSTM	0.853 (0.002)	0.360	0.730 (0.014)	0.463	0.756 (0.014)	0.643
BiComp-DTA	BiComp +FC	L.E+ E.L+ 1DCNN+SepCNN	0.855 (0.002)	0.386	0.703 (0.015)	0.489	0.809 (0.012)	0.598

SW, L.E, E.L, FC, 1DCNN, SepCNN, GNN, FF, and BiLSTM stand for Smith-Waterman similarity, label encoding, embedding layer, fully-connected block, one-dimensional convolutional neural networks, separable CNN, Graph neural networks, Feedforward layer, and bidirectional LSTM block, respectively.

According to Table 6, BiComp-DTA outperformed all baseline methods for cold-drug-protein setting of DTA prediction, in terms of CI and MSE. Furthermore, BiComp-DTA provided best CI for cold-protein setting, compared to the alternative methods. Hence, BiComp-DTA can be safely used for predicting DTA values for novel drugs and proteins, as well as drug-protein pairs.

**Fig 4:**
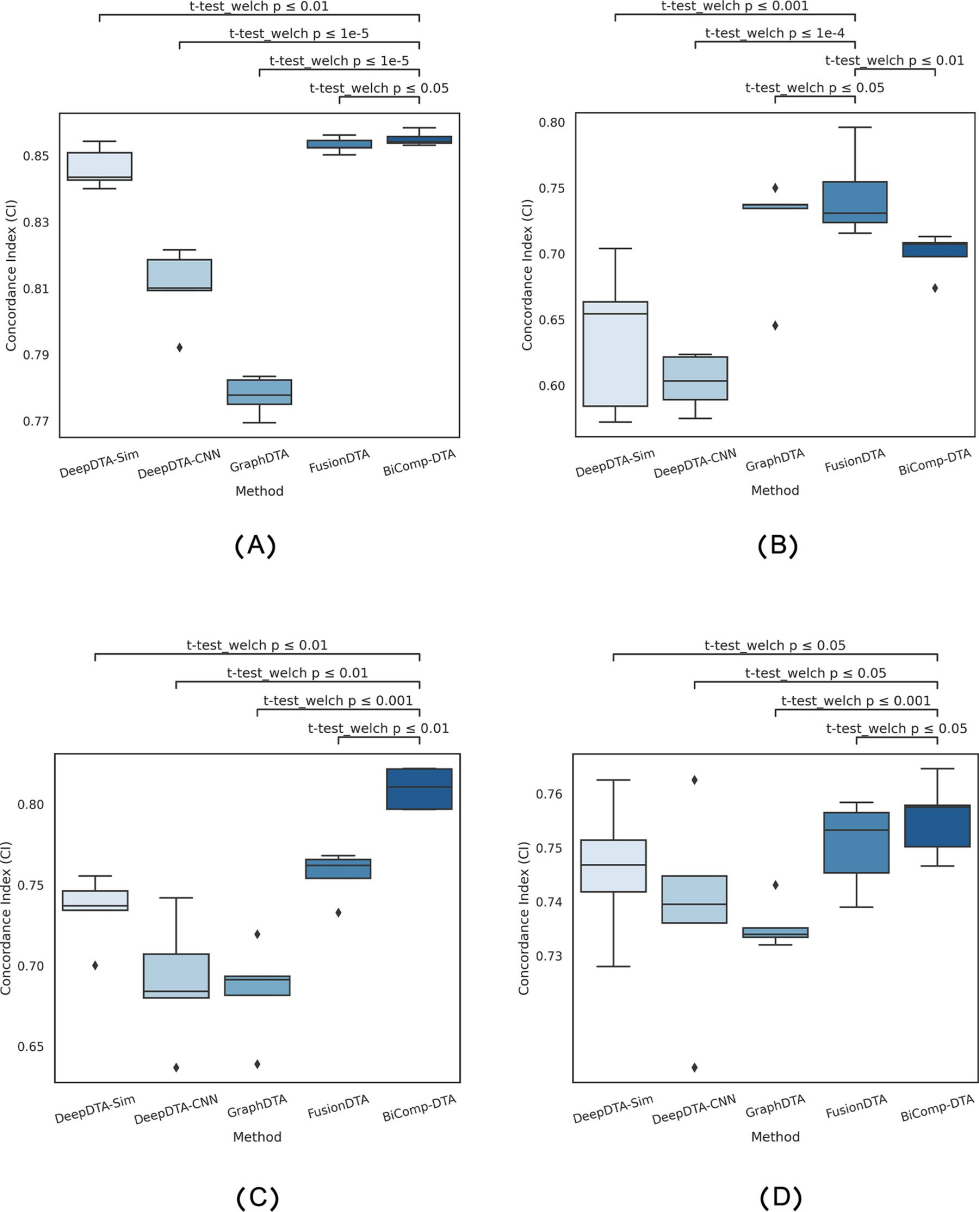
Full distributions of CI values and statistical tests for the BiComp-DTA, compared to the alternative methods, considering alternative splitting settings, (A) cold-protein, (B) cold-drug, and (C) cold-protein-drug settings—Davis dataset, (D) HIV1 protease splitting setting- PDBbind dataset.

Fig 4 represents the distribution of CI values for DeepDTA-Sim, DeepDTA-CNN, GraphDTA, FusionDTA, and BiComp-DTA. The relevant adjusted p-values with Bonferroni corrections for BiComp-DTA and alternative methods on CI are annotated on the plot. According to this figure, the results of the statistical test suggest that BiComp-DTA improves performance prediction by more than 95% and 99% significance for cold-protein and cold-protein-drug experiments, respectively.

To precisely evaluate the ability of the method to extrapolate new data with significant sequence similarity, we considered an alternative splitting setting in the protein family level for the PDBbind dataset. Specifically, we excluded the drug-target pairs including HIV-1 protease variants from the training set and considered them for testing the model. Table 7 provides the comparison of BiComp-DTA, against alternative methods, assuming the protein family splitting setting for the PDBbind dataset. According to this table, BiComp-DTA outperformed all alternative methods for protein family splitting setting for PDBbind dataset in terms of CI and MSE values. The results suggest that BiComp-DTA provided accurate DTA prediction on data with unseen protein sequence for a specific protein variant. Fig 4 represents the distribution of CI values for DeepDTA-Sim, DeepDTA-CNN, GraphDTA, FusionDTA, and BiComp-DTA for protein family splitting experiments. The relevant adjusted p-values with Bonferroni corrections for BiComp-DTA, against alternative methods, on CI are annotated on the plot. According to this figure, the results for statistical test suggest that BiComp-DTA improved performance prediction with above 95% significance for protein family splitting experiments.

**Table 7:** The CI and MSE values for the BiComp-DTA, compared to the alternative methods, assuming protein family splitting setting- PDBbind dataset.

	Proteins	Drugs	CI(std)	MSE
DeepDTA-Sim	SW	L.E+ E.L+ 1DCNN	0.746 (0.012)	1.809
DeepDTA-CNN	L.E+ E.L+ 1DCNN	L.E+ E.L+ 1DCNN	0.738 (0.019)	2.172
GraphDTA	L.E+ E.L+ 1DCNN	GNN	0.736 (0.004)	2.341
FusionDTA	Transformers + FF+ BiLSTM	FF+ BiLSTM	0.751 (0.008)	2.096
BiComp-DTA	BiComp +FC	L.E+ E.L+ 1DCNN+SepCNN	0.756 (0.007)	1.805

SW, L.E, E.L, FC, 1DCNN, SepCNN, GNN, FF, and BiLSTM stand for Smith-Waterman similarity, label encoding, embedding layer, fully-connected block, one-dimensional convolutional neural networks, separable CNN, Graph neural networks, Feedforward layer, and bidirectional LSTM block, respectively.

### 2.5 Adversarial controls for BiComp-DTA performance

**Fig 5:**
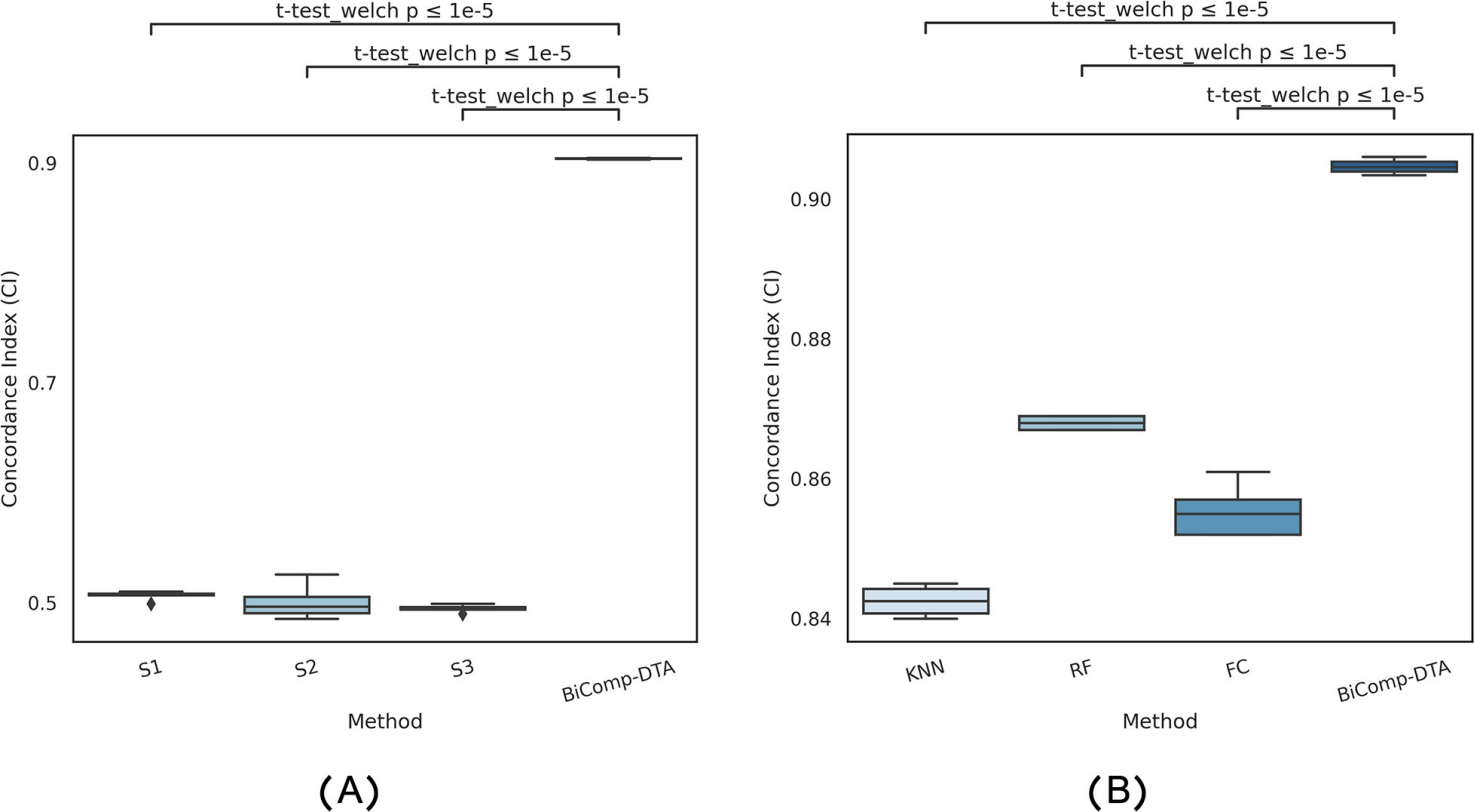
Full distribution of CI values and statistical test for the BiComp-DTA, compared to the (A) straw models S1, S2, S3, and (B) baseline models KNN, RF, and FC- Davis dataset.

For more verification of BiComp-DTA, in this section, we provided a set of adversarial controls experiments, as recommended and applied in [45,46], to ensure that our models do not fit confounding variables and data artifacts. For this purpose, first of all, we trained the straw models on data with shuffled affinity values using three different settings, named S1, S2, and S3 settings. In S1, we trained and tested the models using the shuffled binding affinity values, for which, all the training and testing folds are shuffled. In S2, we performed model training using the shuffled binding affinity values, while unshuffled binding affinity values are used for model testing. Finally, in S3, the test folds are shuffled, while the training folds are unshuffled. Table 8 provides the CI and MSE values, as well as the complete loss of the models in terms of the drop of CI and the increment of MSE, compared to the BiComp-DTA, for three settings for the Davis dataset. According to this table, all experiments on S1, S2, and S3 settings provided CI values near 0.5 and the significant increment of MSE values. Moreover, based on the CI values, we can conclude that for all three settings, the models completely loose the DTA prediction’s performance. The distribution of CI scores and the statistical tests for BiComp-DTA, considering S1, S2, and S3 models, are shown in Fig 5. According to the relevant adjusted p-values on CI values annotated on the plot, BiComp-DTA improved performance prediction with 99% significance for the straw models experiments.

**Table 8:** Straw and baselines models experiment on BiComp-DTA for the Davis dataset.

Experiments		CI(std)	MSE	CI loss	MSE loss
Straw models	S1	0.506 (0.004)	0.794	44%	0.557
	S2	0.501 (0.015)	0.811	44%	0.574
	S3	0.494 (0.003)	1.289	44%	1.052
Baselines	KNN	0.845 (0.002)	0.364	6%	0.127
	RF	0.868 (0.001)	0.317	3%	0.080
	FC	0.855 (0.003)	2.790	5%	2.553
BiComp-DTA		0.904 (0.001)	0.237	-	-

S1, S2, and S3 denote different models, for which, the model training and testing are performed on the shuffled binding affinity values, the model training is performed on the shuffled binding values, and the model testing is performed on the shuffled binding values, respectively. KNN, RF, and FC denote k-nearest neighbors algorithm, random forests, and fully-connected neural network, respectively

Second, to represent the predictive power of BiComp-DTA, we compared our method against three simple baselines, including k-nearest neighbors algorithm, random forests, and fully-connected neural network, for the DTA regression task. In this manner, Table 8 represents CI and MSE values for KNN, RF, and FC, as well as the loss of CI and the MSE increment, compared to the BiComp-DTA. According to this table, BiComp-DTA significantly outperformed these three baseline models, in terms of CI and MSE. The distribution of CI scores and the statistical tests for BiComp-DTA, compared to KNN, RF, and FC models, are shown in Fig 5. According to the relevant adjusted p-values on CI values, annotated on the plot, BiComp-DTA improved performance prediction with above 99% significance for the baseline models experiments.

Third, to explore the ability of BiComp measure to provide an accurate DTA prediction, as well as to investigate the contribution and orthogonality of SW and LZMA measures, we performed two feature ablation experiments, as follows. We trained and evaluated BiComp-DTA using encoded proteins by SW and LZMA measures (i.e. feature ablation SW and feature ablation LZMA), separately. Table 9 provides the comparison results for Davis and Kiba datasets, in terms of four accuracy metrics, CI, MSE, $r_{m}^{2}$, and AUPR. According to this table, BiComp outperformed the SW and LZMA in terms of all four metrics for both datasets. Moreover, LZMA outperformed the SW for Davis dataset, while SW provided better performance, compared to the LZMA, for Kiba dataset. The results suggest that the protein sequence encoding, based on LZMA, may be suitable for datasets with more and longer protein sequences (i.e. Davis dataset), while SW provides better performance for datasets with limited and short protein sequences (i.e. Kiba dataset).

**Table 9:** Performance comparison of BiComp encoding, against LZMA and SW encodings, for drug-target binding affinity prediction, for Davis and Kiba datasets, using feature ablation experiments.

Experiments	Davis				Kiba			
	CI(std)	MSE	$r_{m}^{2}$(std)	AUPR(std)	CI(std)	MSE	$r_{m}^{2}$(std)	AUPR(std)
Feature ablation SW	0.869 (0.001)	0.284	0.541 (0.014)	0.623 (0.005)	0.886 (0.001)	0.168	0.713 (0.016)	0.812 (0.004)
Feature ablation LZMA	0.885 (0.001)	0.279	0.597 (0.015)	0.681 (0.003)	0.877 (0.002)	0.177	0.693 (0.014)	0.803 (0.005)
BiComp-DTA	0.904 (0.001)	0.237	0.696 (0.012)	0.753 (0.006)	0.891(<0.001)	0.167	0.757 (0.012)	0.834 (0.003)

Values of CI, MSE, $r_{m}^{2}$ and AUPR are provided for BiComp compared to those of LZMA and SW encodin*gs for Davis and Kiba datasets*.

**Fig 6:**
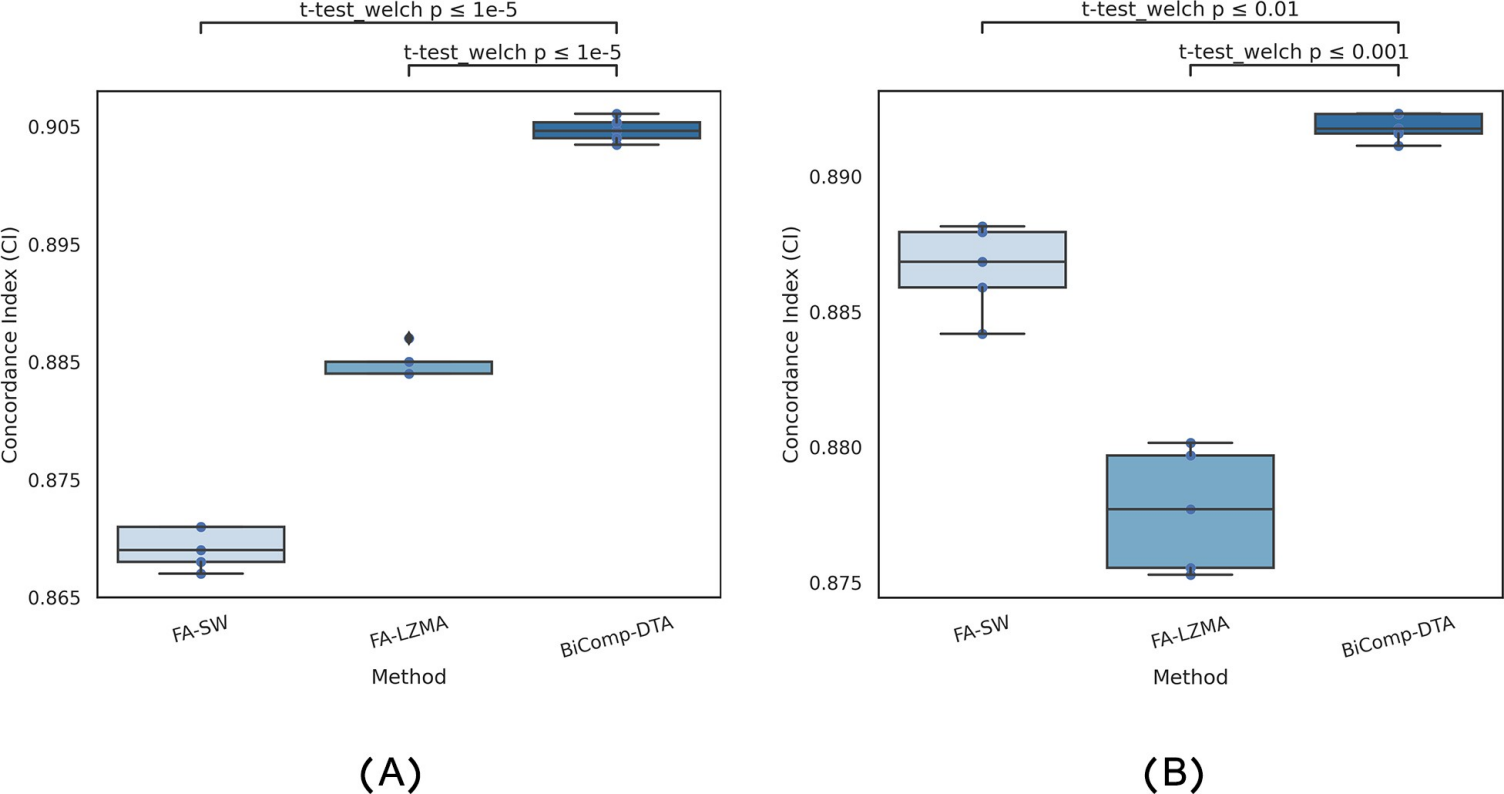
Full distribution of CI values and the statistical test for the BiComp-DTA, compared to the feature ablation SW and lzma- experiments on (A) Davis and (B) Kiba datasets.

The distribution of CI scores and the statistical tests for BiComp-DTA and the feature ablation SW and LZMA (i.e. FA-SW and FA-LZMA) experiments are shown in Fig 6. According to the relevant adjusted p-values on CI values, annotated on the plot, BiComp-DTA improved performance prediction with 99% significance for the feature ablation experiments for Davis and Kiba datasets.

### 2.6 Comparing BiComp-DTA to baselines in terms of the network complexity

Although, some alternative methods have reported outstanding accuracy results for DTA, as discussed in the introduction, they suffer from the complex input models, which requires multiple source of inputs, extra deep models, and layers of representation learning of protein and drug sequences. For a quantitative comparison, we compared the network complexity of BiComp-DTA against that of the GNN-based and Transformer-based DTA methods, in terms of the number of trainable parameters. For better comparisons, a simple fully-connected neural network (FC) is considered which employs encoded drugs in SMILES format based on label-encoding and encoded protein sequences based on BiComp. For this purpose, we compared three implementations of BiComp-DTA, including BiComp-DTA (128, 8), BiComp-DTA (32, 16), and BiComp-DTA (32, 8), where the numbers in brackets indicate the number and the length of filters, respectively, against GraphDTA, FusionDTA, and FC. Table 10 represents the comparison results, in terms of the number of trainable parameters, and the corresponding accuracy values for Davis and Kiba datasets.

**Table 10:** Comparing the BiComp-DTA against the alternative methods, i.e. GraphDTA, FusionDTA, and FC, in terms of the number of trainable parameters, and the corresponding accuracy values for Davis and Kiba datasets (CI).

Method	Number of trainable parameters	Accuracy for Davis dataset (CI)	Accuracy for Kiba dataset (CI)
GraphDTA	4749573	0.880	0.879
FusionDTA	5362081	0.903	0.890
BiComp-DTA (128–8)	2632397	0.904	0.891
BiComp-DTA (32–16)	1948813	0.903	0.887
BiComp-DTA (32–8)	1899149	0.902	0.884
FC	1912833	0.855	0.804

The numbers in parentheses indicate the number and the length of filters for our method, respectively. The number of trainable parameters for GraphDTA and FusionDTA are reported in [24].

According to Table 10, BiComp-DTA provided comparable accuracy for DTA prediction, compared to the GraphDTA and FusionDTA, while preserving light-weight network, in terms of the number of trainable parameters. Specifically, BiComp-DTA (128, 8) provided better performance, compared to the GraphDTA, and FusionDTA for two benchmark datasets with approximately 44%, and 50% fewer number of parameters, respectively. Moreover, although the FC includes fewer number of parameters compared to BiComp-DTA (128, 8), the FC-based network provided poor performance in terms of prediction accuracy. Hence, BiComp-DTA can be more affordable for the massive-scale datasets, while more complicated methods have shown almost comparable accuracy at the cost of higher model complexity.

### 2.7 Comparing BiComp-DTA to baselines in terms of the runtime

Runtime of the BiComp-DTA is compared against the alternative prediction methods on GPUs and CPUs in terms of training time and inference time. For this purpose, execution times of three different implementations of the BiComp-DTA method are compared against runtimes of a simple fully-connected neural network (FC), which employs encoded drugs in SMILES format based on label-encoding and encoded protein sequences based on BiComp, DeepDTA, WideDTA, GraphDTA and FusionDTA. DeepDTA employs label-encoding and CNNs for protein encoding and feature extraction, while WideDTA utilizes extra input sequences and extra CNN block for learning the protein representations. FusionDTA employs ESM-1b transformer [25] along with a fully-connected and a BiLSTM network for distributed protein encoding and feature extraction from the protein. The execution times for training and inference phases are reported in Table 11 for both GPUs and CPUs platforms, in terms of the seconds/epoch. According to this table, all three versions of BiComp-DTA provided smaller training and inference times, compared to the DeepDTA, WideDTA, and FusionDTA, for two benchmark datasets on GPUs and CPUs. BiComp-DTA (128, 8) provided comparable training and inference time, compared to GraphDTA, while BiComp-DTA (32, 16) and BiComp-DTA (32, 8) provided smaller training and inference time, compare to GraphDTA. FC network provided the smallest training and inference time, except for training Kiba dataset on GPUs, at the cost of reduced accuracy. Therefore, BiComp-DTA can be adopted for the massive-scale datasets with large numbers of protein and drug sequences. Furthermore, it can also be adopted for training large datasets on CPUs when GPU resources are limited. In all, BiComp-DTA provides comparable performance, in terms of the prediction accuracy, and a higher speed, compared to the state-of-the-art DTA methods.

**Table 11:** Comparing the BiComp-DTA against the alternative methods, i.e. DeepDTA, WideDTA, GraphDTA, FC, and FusionDTA, in terms of the training and inference times on GPUs and CPUs (seconds/epoch).

Method	GPUs				CPUs			
	Runtime for Davis dataset (seconds/ epoch)		Runtime for Kiba dataset (seconds/epoch)		Runtime for Davis dataset (seconds/ epoch)		Runtime for Kiba dataset (seconds/epoch)	
	Training	Inference	Training	Inference	Training	Inference	Training	Inference
DeepDTA	8	1.1	25	2.9	584	23	1922	87
WideDTA	9	1.4	27	3.5	611	25	2030	88
GraphDTA	2	0.5	13	2	57	4.6	445	42
FusionDTA	137	25	558	101	6207	242	19796	648
BiComp-DTA (128–8)	2	0.7	9	1.5	104	5.3	539	24
BiComp-DTA (32–16)	1	0.5	4	1.3	37	1.7	242	7.8
BiComp-DTA (32–8)	1	0.4	3	1.3	24	1.3	107	5.8
FC	0.01	0.2	7	0.8	1	0.7	26	2.5

The numbers in parentheses indicate the number and the length of filters for our method, respectively.

**Fig 7:**
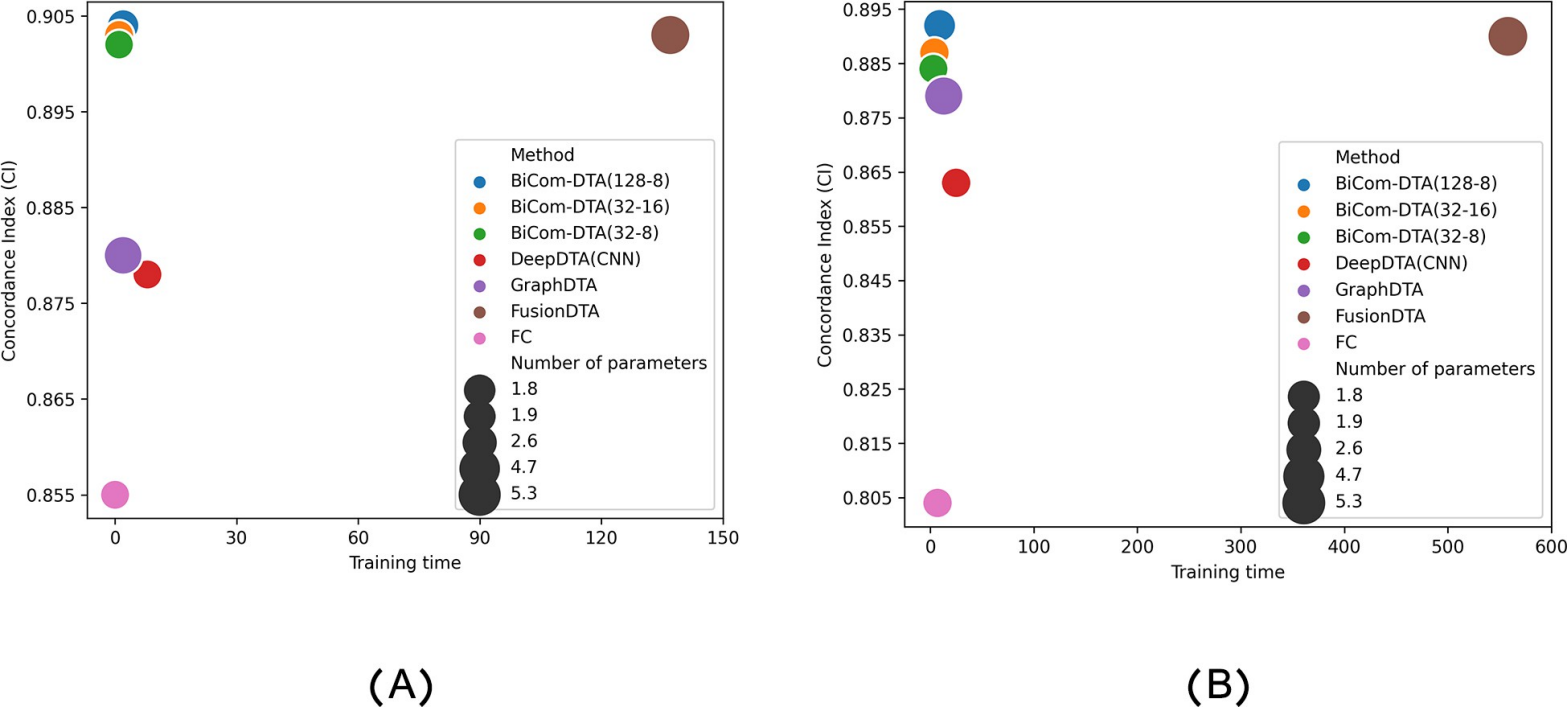
Accuracy vs. training time on GPUs, and the number of parameters for BiComp-DTA and alternative methods. (A) Training time for Davis dataset, and (B) Training time for Kiba dataset.

**Fig 8:**
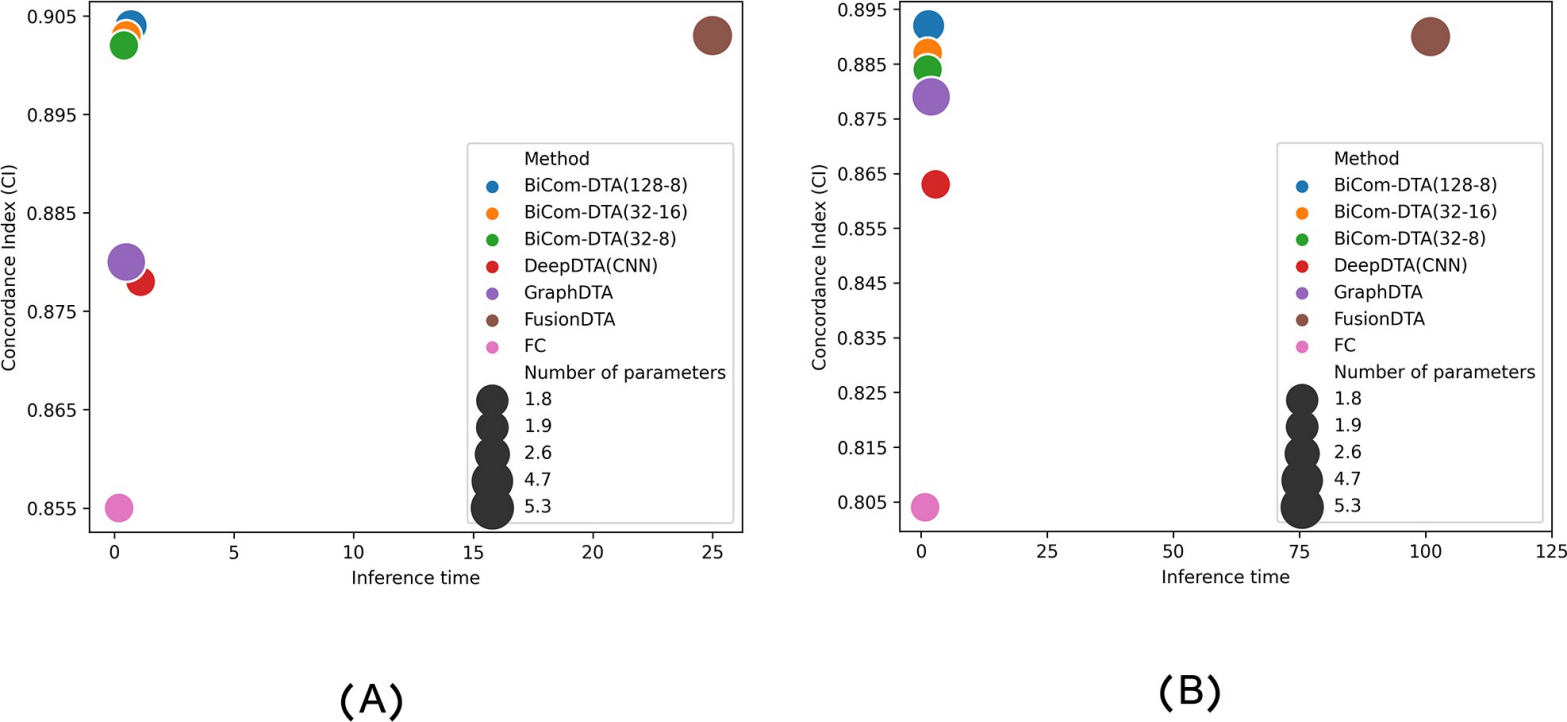
Accuracy vs. inference time on GPUs, and the number of parameters for BiComp-DTA and alternative methods. (A) Inference time for Davis dataset, and (B) Inference time for Kiba dataset.

Moreover, we compared BiComp-DTA and alternative methods in terms of accuracy, runtime, and the number of parameters. According to Figs 7 and 8 BiComp-DTA provided better accuracy (i.e. CI), while preserved the network complexity and runtime for training and inference on GPUs for both the Davis, and Kiba datasets. Furthermore, according to Figs C and D in S1 Text, BiComp-DTA provided similar gains on CPUs. Hence, BiComp-DTA can be considered as a high-performance method in terms of accuracy, and speed with lower processing resources compared to the state-of-the-art DTA methods.

Furthermore, we designed various experiments for investigation the impact of network parameters choices on the BiComp-DTA’s accuracy. Specifically, examining different filters for the CNN block are addressed in Tables B and C in S1 Text, for two benchmark datasets, respectively. Moreover, we evaluated various weighted sums of biological-related and compression-based information for calculating BiComp measure, as reported in Tables D and E in S1 Text, for Davis and Kiba datasets, respectively. Furthermore, we investigated the impact of employing the separable CNN layer along with the CNN layers, on the accuracy, the number of trainable parameters, and the training time as shown in Tables F and G in S1 Text.

## 3. Discussion

In this section, we discuss and analyze the proposed unified measure for protein encoding, as well as the proposed method for DTA prediction. Furthermore, we compare BiComp-DTA against baseline methods in more details, in terms of the prediction accuracy, and the network structure and complexities.

The existing state of the art methods for drug-target binding affinity prediction, which improve the accuracy, utilize multiple sources of input data, very deep and complex neural networks, and extra deep models for feature extraction from protein and drug sequences. Hence adopting those methods for the prediction task enforces data preprocessing, requires domain expert knowledges, and leads to runtime overheads for extra preprocessing, training, and validation. Compared to the alternative methods, BiComp-DTA utilizes a new unified measure for comprehensive and efficient protein encoding, as well as a simple neural network for feature extraction from the encoded protein and drug sequences. Specifically, BiComp measure is proposed for the protein encoding, while a CNN blocks along with a separable CNN layer are adopted for learning the drug representation. The former provides an efficient encoding and avoids multiple sources of input data and the corresponding complicated networks for feature extraction from the protein sequences. The measure has been constructed based on the widely-used Smith-Waterman and the normalized compression distance algorithms. It provides supplementary co-evolution and content-based information for protein sequences in a unified form. Utilizing the unified measure for DTA, we can avoid the extra networks usually required for separate sources of information, and so, prevent the network complexity.

Compared to the similarity-based methods adopting a machine learning algorithm, such as SimBoost, KronRLS, and Sim-CNN-DTA, our method provides a more accurate prediction. Unlike the similarity-based methods, which utilize similarity information for both drugs and targets, BiComp-DTA utilizes sequence-based features and unified similarity-based features for drug and protein sequences, respectively. It is worth noting that some other similarity-based methods, including [11] and NerLTR-DTA, utilize auxiliary information, and need extra efforts and time for preprocessing and constructing multiple high-dimensional matrices. However, BiComp-DTA utilizes a protein similarity matrix taking advantages of the proposed measure with no need to the auxiliary information, such as interaction profile and protein-protein sharing matrix.

Unlike the well-known sequence-based methods, such as DeepDTA, WideDTA, AttentionDTA, and FusionDTA, which employ neural networks on raw protein sequences, our method utilizes encoded proteins based on the BiComp measure along with a fully-connected network. Hence, in addition to extracting both biological-related and content-based information, the proposed method takes advantage of a reduced-complexity network to recover the information loss in the encoded system. Furthermore, BiComp-DTA performs the learning representation stage without extra attention mechanisms, as used in FusionDTA. Hence, it provides comparable accuracy with simple network, in terms of the number of trainable parameters.

Compared to the transformer-based methods, such as MT-DTI and FusionDTA, BiComp-DTA applies simpler inputs and smaller numbers of layers, as well as the trainable parameters for learning the protein and drug representations. Furthermore, unlike the transformer-based methods for the DTA prediction, no fine-tuning stage is required for the BiComp-DTA, while it provides comparable efficiency in terms of the prediction accuracy and the network complexity.

Unlike the GNN-based methods, BiComp-DTA predicts the binding affinities without utilizing external tools for constructing and modeling protein and drug graphs. Specifically, BiComp-DTA does not extract atomic features from the drug compounds, protein contact maps, and drug and protein graph features, and so, reduce the computational time and memory requirement, compared to the GNN-based methods for DTA prediction.

In all, BiComp-DTA provides accurate prediction for drug-target binding affinity prediction with reduced computational complexities, including complexity of the deep neural network, the number of data sources, and the number of trainable network parameters. These achievements basically rely on three main elements or aspects of the proposed method. First of all, the protein sequence encoding using the unified encoding scheme (i.e. BiComp) provides two orthogonal sources of information (co-evolution and content-based information), as a key factor. In this manner, the effectiveness of BiComp and the supplementary contribution of each part (i.e. SW and LZMA), for DTA prediction, have been addressed using feature ablation experiments in Section Results. Second, utilizing a simple but effective FC block for information loss recovery, through the protein sequence encoding process, provides efficient learned features from the encoded protein sequences for the prediction network. And third, employing a CNN block, enhanced with a separable convolutional layer, automatically extracts features from the ligands, in SMILES format, for the prediction network.

## 4. Material and methods

### 4.1 Datasets

We evaluated BiComp-DTA using two available datasets for drug-target binding affinity prediction, known as Davis [40] and Kiba [41] datasets. These two widely-used datasets have been considered as the benchmark in previous works as well. Davis dataset contains the binding affinity values measured by the kinase dissociation constant (Kd), for 68 kinase inhibitor compounds with 442 target proteins. For a more stable training [42], we transformed the binding affinities (i.e. Kd values) into the corresponding logarithmic values (i.e. pKd values), as performed in previous works [10,15,17,18], in a manner illustrated in Eq (1).


$${pK}_{d}=-{log}_{10}(\frac{K_{d}}{1e9})$$ (1)

Kiba dataset includes the binding affinities for 2111 drugs and 229 protein targets. For the Kiba dataset, the affinity values measured by the Kiba scores are captured by several bioactivity information, including the inhibition constant (Ki), the dissociation constant (Kd), and the half maximal inhibitory concentration (IC50).

Moreover, we considered two recently updated datasets, known as BindingDB [42] and PDBbind [43] datasets. Among three versions of BindingDB dataset, which includes the affinity values measured by Kd, Ki, and IC50, we utilized the Kd version of the dataset which includes the binding affinity values for 10665 drug-like small molecules and 1413 protein targets [42]. Compared to the Ki and IC50 versions of the BindingDB dataset, the Kd version includes more binding affinity data with respect to its number of drug-like molecules and protein targets. We considered the maximum affinity values for drug-target pairs, assuming the same sequence information with different binding affinity values as recommended for more data harmonization [42]. The final refined version of BindingDB dataset includes the binding affinities measured by Kd for 9864 drug-like small molecules and 1088 protein targets. For more stable training, we transformed the binding affinities (i.e. Kd values) into the corresponding logarithmic values (i.e. pKd values) [42].

The PDBbind dataset includes experimentally measured binding affinity data for drug-target complexes deposited in the Protein Data Bank (PDB). From the available subsets of PDBbind version 2020, including the general set, the refined set, and the core sets, we chose the refined set including data with better quality, compared to the general set, and larger dataset, compared to the core set [43]. The refined set includes the binding affinity values, measured by Ki and Kd, and transformed to the log-scale values (i.e. pKi and pKd values) for 4295 drugs and 1606 protein targets. After removing any probable redundancy in drugs with multiple sequences in SMILES format, the final utilized set includes the binding affinity values for 4231 drugs and 1606 protein targets. The summary of the utilized datasets is shown in Table 12, while Figs A and B in S1 Text provide more detailed information for Davis, Kiba, BindingDB, and PDBbind datasets.

**Table 12:** The summary of benchmark datasets.

	Proteins	Compounds	Interactions	Affinity measure
Davis	442	68	30056	Dissociation constant(Kd)
Kiba	229	2111	118254	Inhibition constant(Ki) Dissociation constant(Kd) Half-maximal inhibitory concentration(IC50)
BindingDB	1088	9864	42203	Dissociation constant(Kd)
PDBbind	1606	4231	5014	Inhibition constant(Ki) Dissociation constant(Kd)

### 4.2 BiComp-DTA method

**Fig 9:**
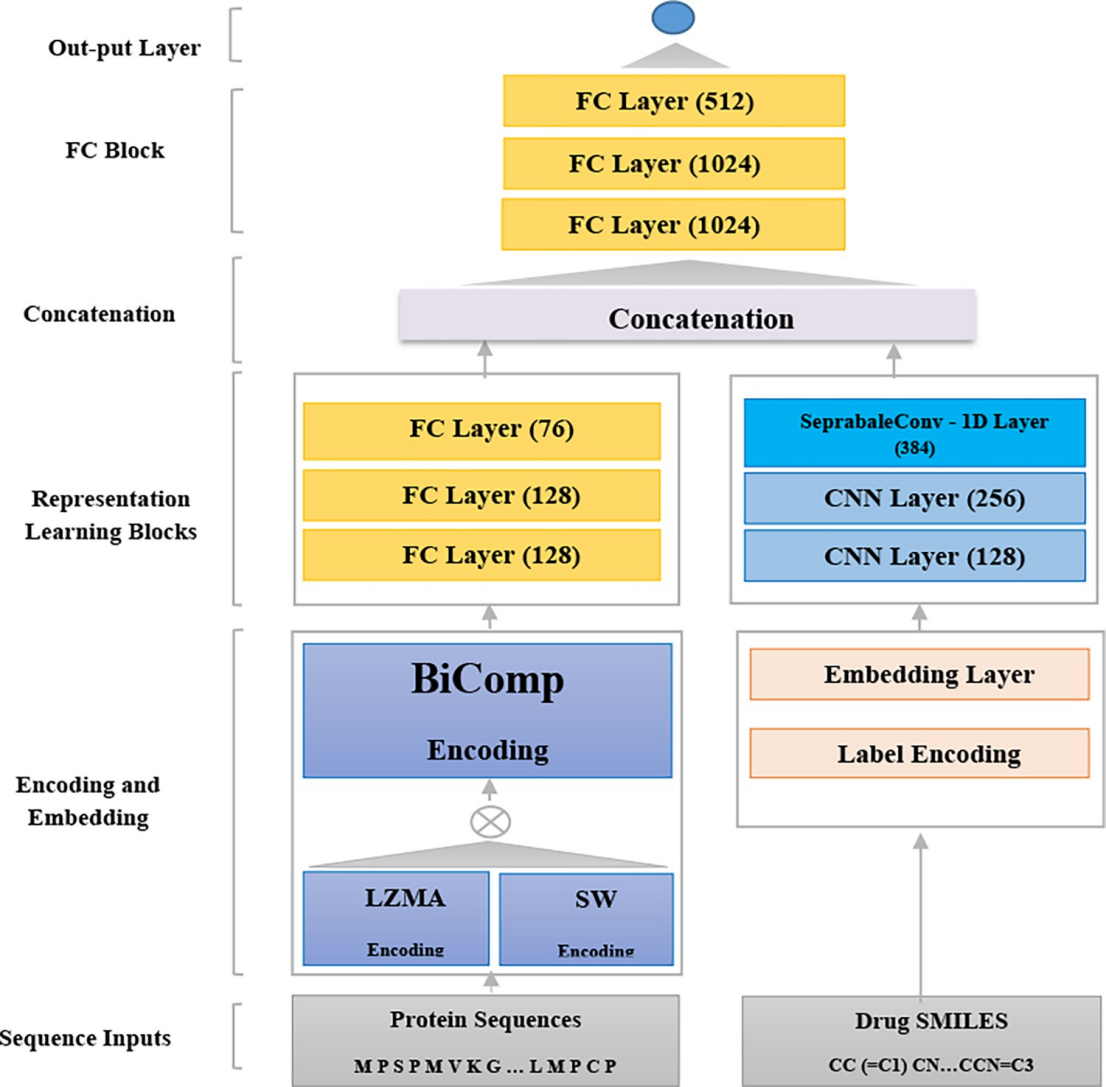
Overview of BiComp-DTA method for drug-target binding affinity prediction. The protein sequences, are encoded using the BiComp measure to capture biological-related and compression-based information. The encoded proteins are passed to a fully-connected block with a specific number of neurons, as represented in the parentheses, for more information loss recovery through the encoding process. For the drug sequences in SMILES format, we adopt widely-used simple label encoding, utilized in the prior studies. The encoded SMILES sequences are passed to an embedding layer, along with a CNN block including two CNN and one separable CNN layers with the specific numbers of filters, as represented in brackets, and a max-pooling layer following the separable CNN layer. For an efficient feature extraction form the drug sequences, we employ a separable convolutional layer, to achieve accurate representation learning with fewer trainable parameters. Learnt representations of proteins and drugs sequences are concatenated and passed to a three layer fully-connected block, as the predictor, followed by an output layer.

Our proposed method for drug-target binding affinity prediction, named BiComp-DTA, includes four units: a) data encoder, b) feature extractor, c) concatenator, and d) predictor. The overall method is illustrated in Fig 9.

### A) Data encoder

As the first step of BiComp-DTA method, the input ligands, in SMILES format, and the input protein sequences are encoded using the widely-used label-encoding and the proposed unified measure, respectively. To encode the ligands in SMILES format, we assign a unique integer value to each character. It should be noted that due to the varying length of ligands in SMILES format, we choose a fixed maximum length of 85, 100, 200, and 200 for SMILES for Davis, Kiba, BindingDB, and PDBbind datasets, respectively. Therefore, the ligands in SMILES format that are shorter than the maximum length are zero-padded, while the longer ones are truncated. In this manner, each ligand in SMILES format is encoded to the same length integer vector.

To encode the protein sequences, we propose a unified measure, named BiComp, which provides information theory-based features, while preserving important evolutionary-related features from the protein sequences. To calculate the measure, we employ two widely-used algorithms, known as the Smith-Waterman (SW) and the Lempel–Ziv–Markov chain (LZMA) algorithms. The Smith-Waterman, as a well-known algorithm for biological sequence alignment, has been employed to multiple area of computational biology, ranging from the whole-genome alignment to the protein sequence comparison [37,38]. We employ the normalized Smith-Waterman (SW) alignment score, as the first measure for calculating the protein sequences similarity. It should be noted that the utilization of normalized version of SW scores provides data in a common scale, as well as a more stable training step. The normalized Smith-Waterman score (*S*_*SW*_(*p*_*i*_, *p*_*j*_)) for each pair of protein sequence *p*_*i*_ and *p*_*j*_ is computed as follows.


$$S_{SW}(p_{i},p_{j})=\frac{SW(p_{i},p_{j})}{\sqrt{SW(p_{i},p_{i})}\sqrt{SW(p_{j},p_{j})}}$$ (2)

SW measure is calculated based on a local sequence alignment algorithm (i.e. Smith-Waterman), which identifies the similar regions, and so, measures the functional, structural, or evolutionary relationships between protein sequences pairs [47]. In this manner, the SW measure can provide various information, ranging from the protein sequence rearrangement-related information, to the sequence homology and structural-related information (implicitly).

On the other hand, the normalized compression distance (NCD) [48,49] provides the similarity between pair of protein sequences using the shared information content [48,50,51]. The measure is calculated based on the Kolmogorov complexity [52] to approximate the normalized information distance for a pair of protein sequences [37]. This measure is selected based on its key advantages as described following. As an alignment-free measure, the NCD is independent from many evolutionary events, such as rearrangements. Moreover, assuming contiguity of the conserved regions [37,49] facilitates mutual information extraction from the sequences [49]. The normalized compression distance *NCD*(*p*_*i*_, *p*_*j*_) for each pair of protein sequences *p*_*i*_ and *p*_*j*_ is calculated as follows [48].


$$NCD(p_{i},p_{j})=\frac{C(p_{i}p_{j})-min\{C(p_{i}),C(p_{j})\}}{max\{C(p_{i}),C(p_{j})\}}$$ (3)

Here, *C*(*p*_*i*_) and *p*_*i*_*p*_*j*_ represent the length of compressed sequence *p*_*i*_ and the concatenation result of sequences *p*_*i*_ and *p*_*j*_, respectively.

The NCD measure provides the content distance between two protein sequences, where the higher distance values indicate a lower pairwise similarity. The latter measure, named *S*_*NCD*_(*p*_*i*_, *p*_*j*_), can be calculated for each pair of protein sequences *p*_*i*_ and *p*_*j*_ according to Eq (4).


$$S_{NCD}(p_{i},p_{j})=1-NCD(p_{i},p_{j})$$ (4)

For calculating the similarity between protein sequences, based on NCD measure, we employ the Lempel–Ziv–Markov chain algorithm (LZMA), which provides lossless data compression, as adopted for biological applications, such as sequence classification [37,38].

Summarizing above discussion, we believe that the SW and NCD measures can provide complementary information for encoding the protein sequences to feed a drug-target binding affinity predictor. For this purpose, a new unified measure is proposed in this paper, named BiComp which is calculated as follows.


$$BiComp(p_{i},p_{j})=S_{NCD}(p_{i},p_{j})\times S_{SW}(p_{i},p_{j})$$ (5)

The protein encoding unit utilizing BiComp performs following three major steps.

1. *S*_*SW*_ Encoding. At the first step, the first protein similarity matrix is constructed by comparing each pair of protein sequences *p*_*i*_, *p*_*j*_ and producing the normalized SW similarity measure (*S*_*SW*_(*p*_*i*_, *p*_*j*_)). For this purpose, protein sequences are encoded as vectors, with the length of each vector equal to the total number of sequence samples of the corresponding dataset.
2. *S*_*NCD*_ Encoding. At the second step, the second protein similarity matrix is constructed by comparing each pair of protein sequences *p*_*i*_, *p*_*j*_, and producing the normalized compression similarity measure (*S*_*NCD*_(*p*_*i*_, *p*_*j*_)). In this manner, protein sequences are encoded as vectors, with the length of each vector equal to the total number of sequence samples of the corresponding dataset.
3. BiComp Encoding. At the final step, the final encoding matrix, BiComp is generated by the Hadamard product [53,54] of the SW and NCD similarity matrices.

### B) Feature extractor

As the second step of the BiComp-DTA method, the encoded ligands and proteins sequences are passed to the corresponding neural networks for feature extraction. For protein feature extraction, a three layer fully-connected neural network is employed, while for learning the ligand representation, we propose a new neural network architecture with two CNN layers followed by a separable CNN layer. The separable CNN performs a depth-wise convolution followed by a point-wise convolution, and so, reduces the number of network parameters. Hence, by decreasing the network complexity, the training and inference processing times are improved. It is worth noting that to address the trade-off between the network complexity, in terms of the number of parameters, and the network performance, we employ a separable CNN, as the last layer of the drug sequence feature extraction network.

### C) Concatenator

As the third step of the BiComp-DTA method, the representation outputs from the feature extractor for ligands and proteins sequences are concatenated. The merged features are passed to the predictor unit to provide the final prediction.

### D) Predictor

As the last step of the BiComp-DTA method, we employ a three layer fully-connected neural network followed by an output layer to predict the binding affinity values.

## 5. Conclusion and future works

Prediction the affinity values between compounds and protein targets is an important task in drug discovery. In this paper, we propose a unified measure, i.e. BiComp, for protein encoding that includes evolutionary-based and compression-based features for protein encoding in drug-target binding affinity prediction. Furthermore, we proposed a deep learning-based method, i.e. BiComp-DTA, for affordable drug-target binding affinity prediction in terms of accuracy and computational complexities. BiComp-DTA utilizes BiComp measure for efficient protein encoding, provides accurate prediction performance without utilizing various complicated sources of input data and deep neural networks. The proposed measure provides supplementary information in the form of a unified vector, instead of construction and utilizing multiple sources of data for efficient protein encoding. Hence, the preprocessing and feature extraction from protein inputs can be efficient. The proposed method, i.e. BiComp-DTA, utilizes a fully connected network for more accurate feature extraction from the encoded protein and a CNN block including an efficient separable CNN layer for feature extraction from the drug sequences in SMILES format. The results suggest that BiComp-DTA can be applied as an efficient DTA method in terms of binding affinity prediction accuracy as well as simplicity of the overall network architecture. Furthermore, comparison between the proposed measure, i.e. BiComp, against SW and LZMA, suggest the measure can be utilized for DTA prediction along with GNN-based and Transformer-based methods for more accurate and affordable network architecture, as future works. Furthermore, as the future works, we will utilize the proposed measure for various interaction prediction tasks, such as protein-protein interaction prediction, as well as for protein family classification.

## Supporting information

S1 Text Supporting information. **Table A: Parameter settings for BiComp-DTA. Fig A: Affinity values distribution (the pKd values for Davis and the Kiba scores for Kiba datasets) and the lengths of the drug in SMILES format and the protein sequences for Davis and Kiba datasets. Fig B: Affinity values distribution (the pKd values for BindingDB and the pKi, and pKd values for PDBbind datasets) and the lengths of the drug in SMILES format and the protein sequences for BindingDB and PDBbind datasets. Table B: The CI, and MSE values for different filters—Davis dataset. Table C: The CI and MSE values for different filters—Kiba dataset. Table D: The summation weight of biological and compression features to calculate the BiComp measure—Davis dataset. Table E: The summation weight of biological and compression features to calculate the BiComp measure—Kiba dataset. Table F: The results for the model with different position of the separable CNN—Davis dataset. Table G: The results for the model with different position of the separable CNN—Kiba dataset. Fig C: Accuracy vs. training time on CPUs, and the number of parameters for BiComp-DTA and alternative methods.** (A) Training time for Davis dataset, and (B) Training time for Kiba dataset. **Fig D: Accuracy vs. inference time on CPUs, and the number of parameters for BiComp-DTA and alternative methods.** (A) Inference time for Davis dataset, and (B) Inference time for Kiba dataset.
(DOCX)
