# Supplementary material for: BiComp-DTA: Drug-target binding affinity prediction through complementary biological-related and compression-based featurization approach
Source: PLoS Comput Biol. 2023 Mar 31;19(3):e1011036. doi: 10.1371/journal.pcbi.1011036 (PMC10096306; doi:10.1371/journal.pcbi.1011036)
Supplement: S1 Text — Table A: Parameter settings for BiComp-DTA. Fig A: Affinity values distribution (the pKd values for Davis and the Kiba scores for Kiba datasets) and the lengths of the drug in SMILES format and the protein sequences for Davis and Kiba datasets. Fig B: Affinity values distribution (the pKd values for BindingDB and the pKi, and pKd values for PDBbind datasets) and the lengths of the drug in SMILES format and the protein sequences for BindingDB and PDBbind datasets. Table B: The CI, and MSE values for different filters—Davis dataset. Table C: The CI and MSE values for different filters—Kiba dataset. Table D: The summation weight of biological and compression features to calculate the BiComp measure—Davis dataset. Table E: The summation weight of biological and compression features to calculate the BiComp measure—Kiba dataset. Table F: The results for the model with different position of the separable CNN—Davis dataset. Table G: The results for the model with different position of the separable CNN—Kiba dataset. Fig C: Accuracy vs. training time on CPUs, and the number of parameters for BiComp-DTA and alternative methods. (A) Training time for Davis dataset, and (B) Training time for Kiba dataset. Fig D: Accuracy vs. inference time on CPUs, and the number of parameters for BiComp-DTA and alternative methods. (A) Inference time for Davis dataset, and (B) Inference time for Kiba dataset. (DOCX) [file pcbi.1011036.s001.docx]

**BiComp-DTA: Drug-target binding affinity prediction through complementary biological-related and compression-based featurization approach**

Mahmood Kalemati^^[[1]](#footnote-1)^1^, Mojtaba Zamani Emani^^[[2]](#footnote-2)^1^ and Somayyeh Koohi^^[[3]](#footnote-3)^1^[[4]](#footnote-4)^*^

Department of Computer Engineering, Sharif University of Technology, Tehran, Iran

^*^ Corresponding author

E-mail: koohi@sharif.edu (SK)

**Supporting information**

# Evaluation metrics

To evaluate the performance of our binding prediction method, named BiComp-DTA, and our unified measure for protein encoding, named BiComp, we use four widely-used metrics, including Concordance Index (CI), Mean Squarer Error (MSE), $r_{m}^{2}$ , and AUPR.

The first metric, CI, is defined by Equation (1), and indicates the prediction performance of a regression model [‎1], where $f_{i}$ and $f_{j}$ are the predicted values for the ground truth affinity values $y_{i}$ and $y_{j}$ ($y_{i}>y_{j}$), respectively.

$CI=\frac{1}{z}\sum_{y_{i}>y_{j}} h\left( f_{i}-f_{j} \right)$ (1)

Here, Z stands for the normalization constant, while h as a step function is given by Equation (2).

$h\left( x \right)=\left\{ \begin{aligned} 1, if x>0 \\ 0.5, if x=0 \\ 0, if x<0 \end{aligned} \right.$ (2)

The concordance index, also known as the c-index, is actually a ranking metric which determines if the predicted and the actual affinity values for two random drug-target pairs are in the correct order. In this manner, the c-index value equal to 1 and 0 for the best and random prediction, respectively.

Equation (3) defines the second evaluation metric, MSE, to measure the difference between the actual and predicted values of binding affinity. Here, n stands for the number of samples, and P and Y are the predicted and the true affinity values, respectively.

$MSE=\frac{1}{n}\sum_{i=1}^{n} \left( P_{i}-Y_{i} \right)^{2}$(3)

The third widely-used metric, $r_{m}^{2}$ , represents the external prediction performance of a quantitative structure-activity relationship (QSAR) model, and is defined according to Equation (4), where $r^{2}$ and $r_{0}^{2}$ stand for the squared correlation coefficients values with and without intercept, respectively [ ‎2, ‎3, ‎4]. In this way, a model is acceptable if $r_{m}^{2}>0.5$.

$r_{m}^{2}= r^{2}\times(1-\sqrt{(r^{2}-r_{0}^{2})})$ (4)

The last metric for evaluating BiComp-DTA, AUPR, indicates the model performance for binary classification. For formulating a classification problem, the affinity values are transformed into the corresponding binary values by considering the threshold values of 7 and 12.1 for Davis and Kiba datasets, respectively, as chosen in prior works [‎5, ‎6, ‎7].

# Implementation details

The development of our method is performed using Keras API [‎8] written in Python and Tensorflow machine learning platform [‎9]. The implementation and performance evaluation of BiComp-DTA on two benchmark datasets is performed on Ubuntu 18.04 with Intel(R) Xeon(R) CPU @ 2.30GHz and NVIDIA’s GeForce GTX 1080 with 11 GB available memory. For the performance evaluation, we used two datasets, known as Davis and Kiba as described in Section Material and Methods. In this end, we adopted 5-fold cross-validation, as conducted in DeepDTA [‎6], where the training and hyper-parameter tuning are performed using five nearly equal size training and validation sets. The parameter settings for the model are illustrated in Table A in S1 Text. Finally, a hold-out test set is considered to provide an unbiased performance evaluation of the final model.

# Datasets details

The affinity values distributions (i.e. the pKd values for Davis and the Kiba scores for Kiba datasets), as well as the lengths of the drug SMILES and the protein sequences for Davis and Kiba datasets, are provided in Fig A in S1 Text. Fig B in S1 Text provides the affinity values distributions (the pKd values for BindingDB and the pKi, and pKd values for PDBbind datasets) and the lengths of the drug in SMILES format and the protein sequences for BindingDB and PDBbind datasets.

**Table**  **A in S1 Text. Parameter settings for BiComp-DTA**

| Length of SMILES(drug) | 85,100,200,200 |
| --- | --- |
| Number of filters(drugs) | 32,128 |
| Filter length(drugs) | 4,8,16 |
| Number of neurons (protein) | 128,128,76 |
| Number of neurons (DTA) | 1024,1024,512 |
| Number of epochs | 250,500,1000,2000 |
| Batch size | 704 |
| Dropout[‎10] | 0.2 |
| Optimizer function | Adam |
| Learning rate | 0.001 |

**(A)**

**(B)**

**(C)**


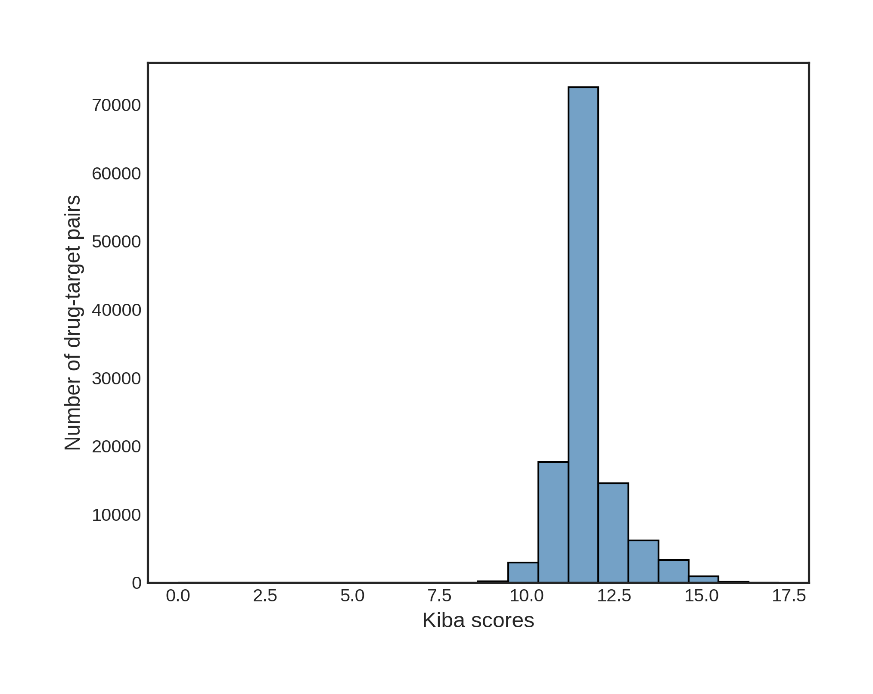

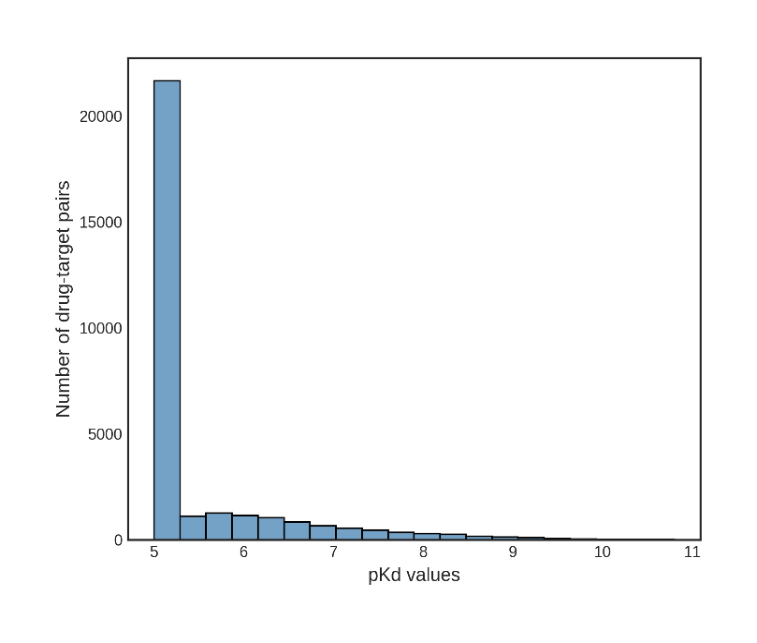

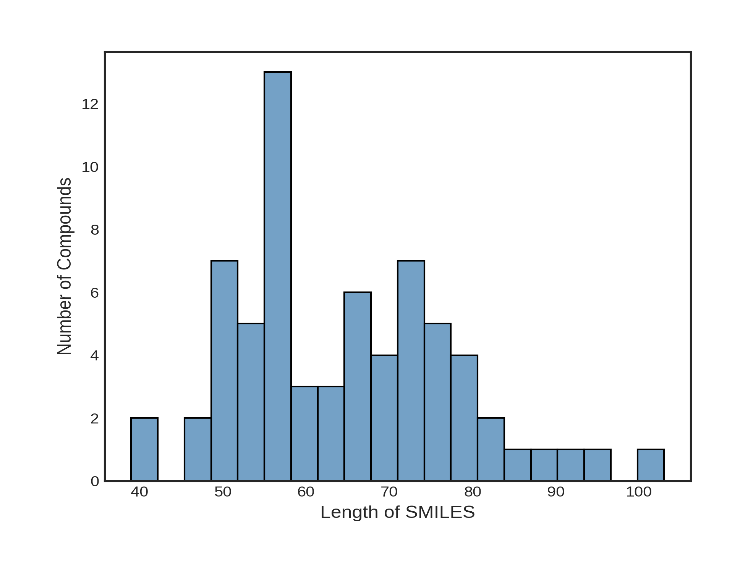

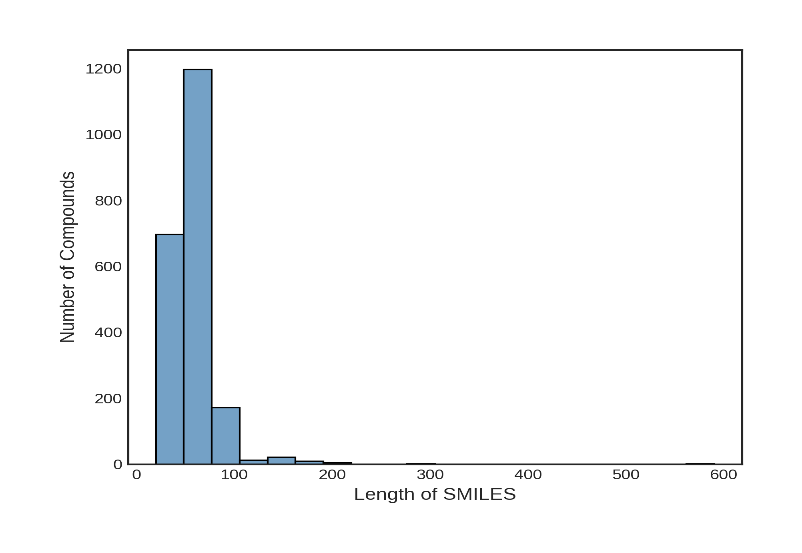

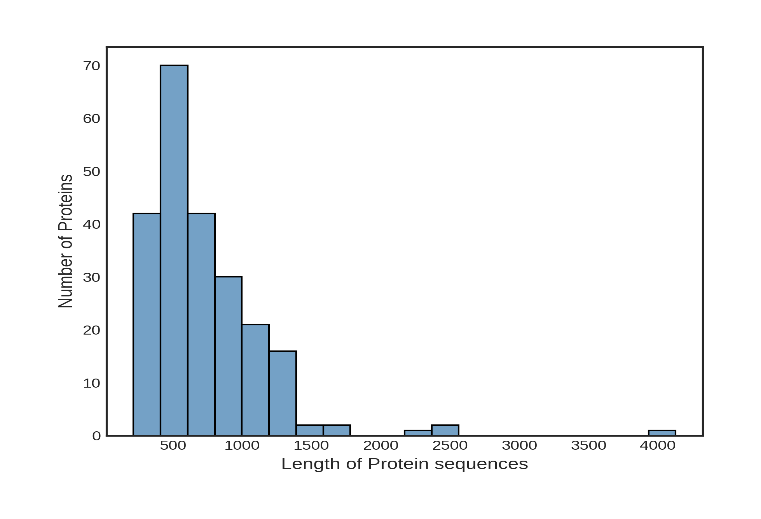

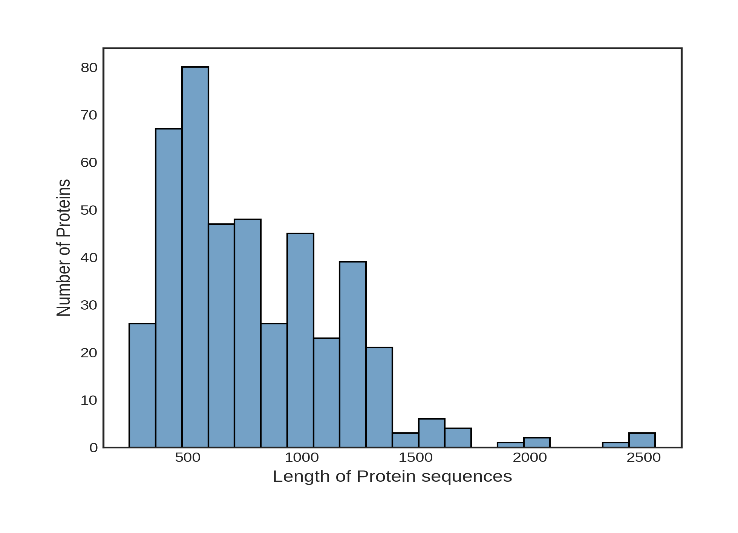


**Fig A in S1 Text.** **Affinity values distribution (the pKd values for Davis and the Kiba scores for Kiba datasets) and the lengths of the drug in SMILES format and the protein sequences for Davis and Kiba datasets**.

**(A)**

**(B)**

**(C)**


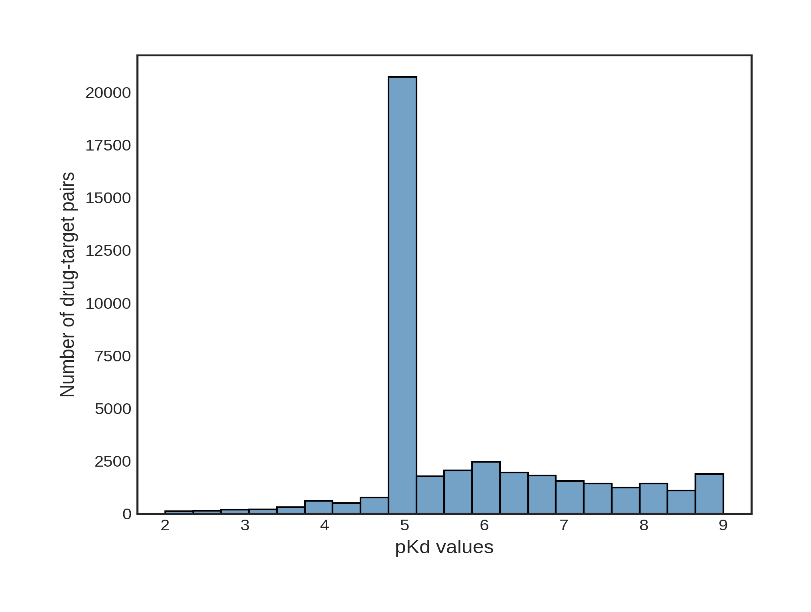

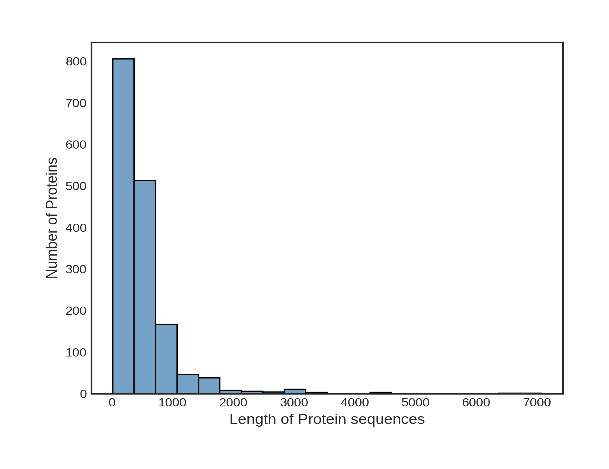

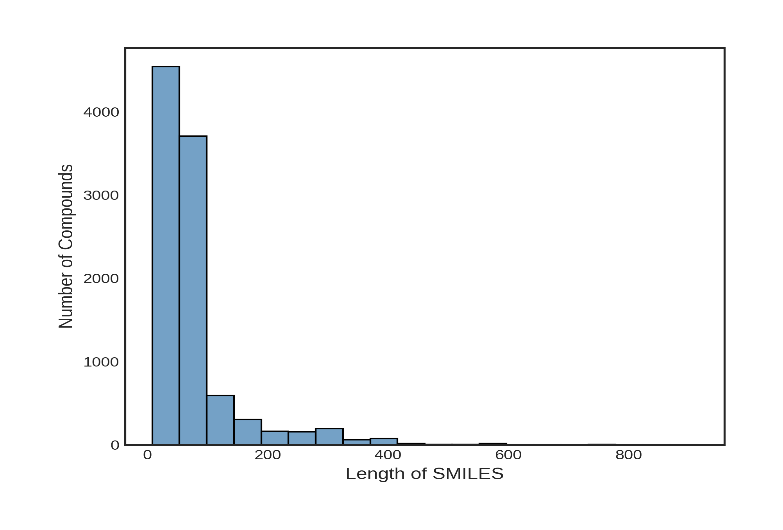

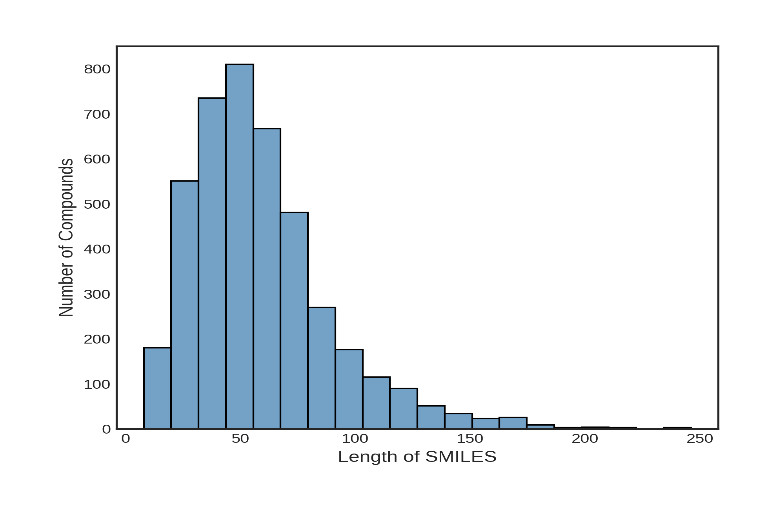

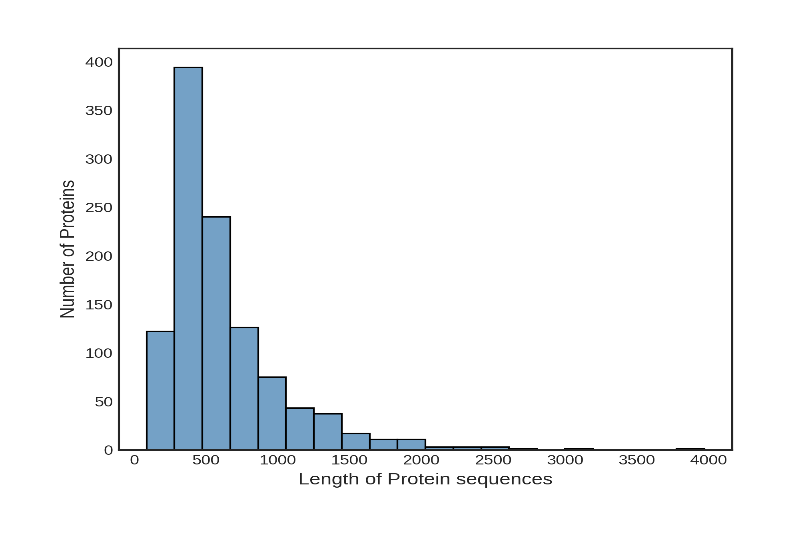

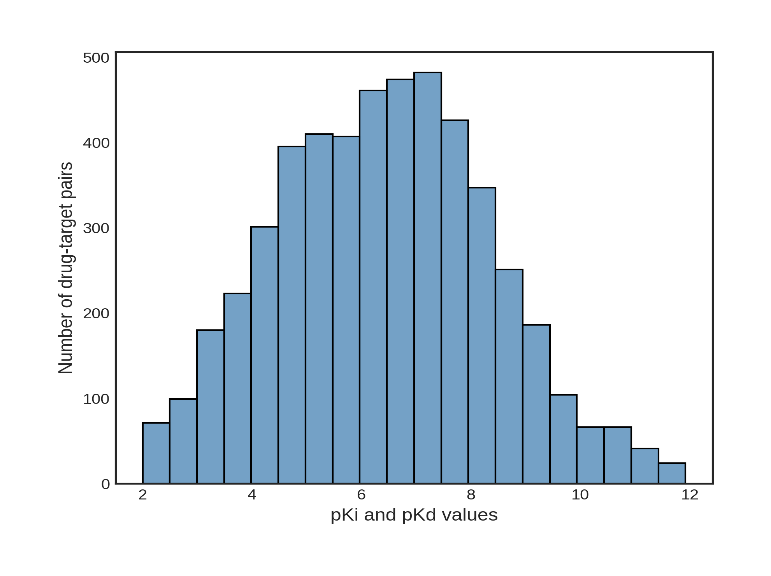


**Fig B in S1 Text.** **Affinity values distribution (the pKd values for BindingDB and the pKi, and pKd values for PDBbind datasets) and the lengths of the drug in SMILES format and the protein sequences for BindingDB and PDBbind datasets**.

# Choosing proper number of filters and their lengths

We investigated employment of different filters for the CNN block, as shown in Tables B and C in S1 Text. These tables represent the CI and MSE values assuming one out of five folds is used for testing various filters for Davis and Kiba datasets, respectively. According to Tables B and C in S1 Text, the number of filters and the length of filters of 128 and 8, respectively, provide the best values of CI and MSE for both two benchmark datasets.

**Table B in S1 Text.** **The CI, and MSE values for different filters - Davis dataset**

| The number of filters | The length of filters | CI | MSE |
| --- | --- | --- | --- |
| 32 | **4** | 0.897 | 0.231 |
| 32 | **8** | 0.901 | 0.224 |
| 32 | **16** | 0.900 | 0.226 |
| 128 | **4** | 0.896 | 0.227 |
| 128 | **8** | 0.902 | 0.225 |
| 128 | **16** | 0.899 | 0.226 |

**Table C in S1 Text. The CI and MSE values for different filters - Kiba dataset**

| The number of filters | The length of filters | CI | MSE |
| --- | --- | --- | --- |
| 32 | **4** | 0.878 | 0.182 |
| 32 | **8** | 0.883 | 0.175 |
| 32 | **16** | 0.884 | 0.173 |
| 128 | **4** | 0.888 | 0.165 |
| 128 | **8** | 0.893 | 0.160 |
| 128 | **16** | 0.890 | 0.165 |

# Summation weights of the biological and compression features for calculating BiComp measure

We conducted various experiments for determining the summation weights for the biological-related and compression-based features to efficiently construct the BiComp measure. Tables D and E in S1 Text represent CI and MSE values assuming one out of five folds is used for testing various weights of LZMA and SW measures for the Davis and Kiba benchmark datasets, respectively.

**Table D in S1 Text.** **The summation weight of biological and compression features to calculate the BiComp measure - Davis dataset**

| LZMA weight | SW weight | CI | MSE |
| --- | --- | --- | --- |
| 0.25 | **1** | 0.886 | 0.244 |
| 1 | **0.25** | 0.887 | 0.241 |
| 0.75 | **0.25** | 0.881 | 0.249 |
| 0.25 | **0.75** | 0.877 | 0.241 |
| 0.75 | **1** | 0.894 | 0.227 |
| 1 | **0.75** | 0.897 | 0.227 |
| 1 | **1** | 0.902 | 0.225 |

**Table E in S1 Text. The summation weight of biological and compression features to calculate the BiComp measure - Kiba dataset**

| LZMA weight | SW weight | CI | MSE |
| --- | --- | --- | --- |
| 0.25 | **1** | 0.885 | 0.165 |
| 1 | **0.25** | 0.885 | 0.164 |
| 0.75 | **0.25** | 0.885 | 0.168 |
| 0.25 | **0.75** | 0.887 | 0.164 |
| 0.75 | **1** | 0.886 | 0.162 |
| 1 | **0.75** | 0.890 | 0.162 |
| 1 | **1** | 0.893 | 0.160 |

According to Tables D and E in S1 Text, equal summation weights for LZMA and SW measure provide the best values of CI and MSE for the two benchmark datasets.

# Adoption of separable CNNs

We investigated the impact of employing the separable CNN layer along with the CNN layers, on the accuracy, the number of trainable parameters, and the training time. For this purpose, we adopted one separable CNN layer, either as the first, second, or the third layer, for learning the drug sequences representation. Tables F and G in S1 Text represent the comparison results, assuming one out of five folds is used for testing these three network choices for Davis and Kiba datasets, respectively.

**Table F in S1 Text.** **The results for the model with different position of the separable CNN - Davis dataset**

| Layer of adopting the separable CNN | CI | MSE | Number of parameter | Time(seconds/epochs) |
| --- | --- | --- | --- | --- |
| 1^st^ layer | 0.893 | 0.236 | 3204813 | 3 |
| 2^nd^ layer | 0.897 | 0.231 | 3090125 | 2 |
| 3^rd^ layer | 0.902 | 0.225 | 2632397 | 2 |

**Table G in S1 Text.** **The results for the model with different position of the separable CNN - Kiba dataset**

| Layer of adopting the separable CNN | CI | MSE | Number of parameter | Time(seconds/epochs) |
| --- | --- | --- | --- | --- |
| 1^st^ layer | 0.887 | 0.164 | 3177549 | 13 |
| 2^nd^ layer | 0.888 | 0.167 | 3062861 | 12 |
| 3^rd^ layer | 0.893 | 0.160 | 2605133 | 10 |

According to Tables F and G in S1 Text, utilizing the separable CNN in the third layer, along with the CNN layers, improves the network performance in terms of accuracy, the number of parameters, and the training time.

# Comparing accuracy, runtime on CPUs, and the number of parameters for BiComp-DTA and alternative methods

We compare BiComp-DTA and alternative methods in terms of accuracy, runtime on CPUs, and the number of parameters. According to Figs C and D in S1 Text, BiComp-DTA provides better accuracy (i.e. CI), while preserving the network complexity and runtime for training and inference on CPUs for both the Davis and Kiba datasets.

**(A)**

**(B)**


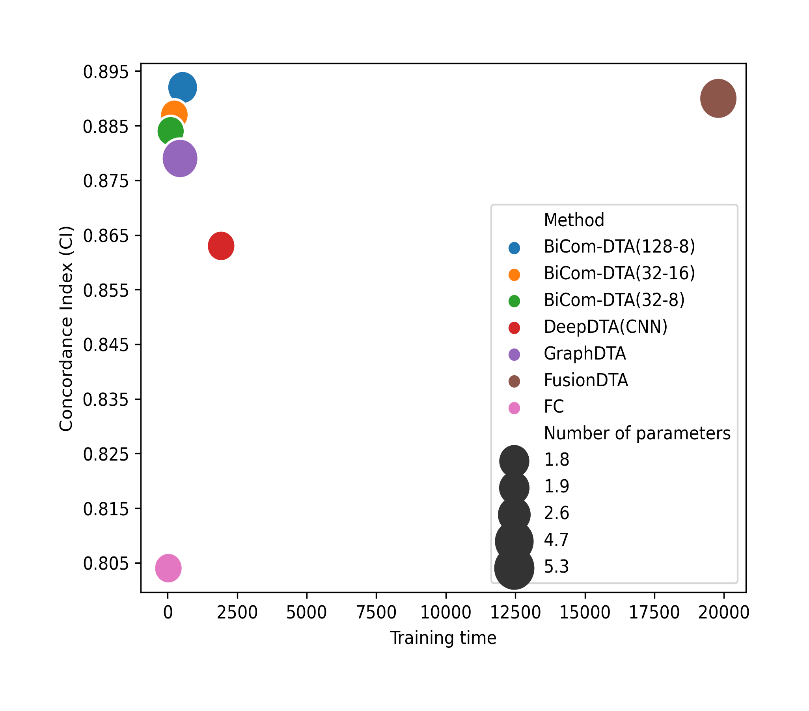

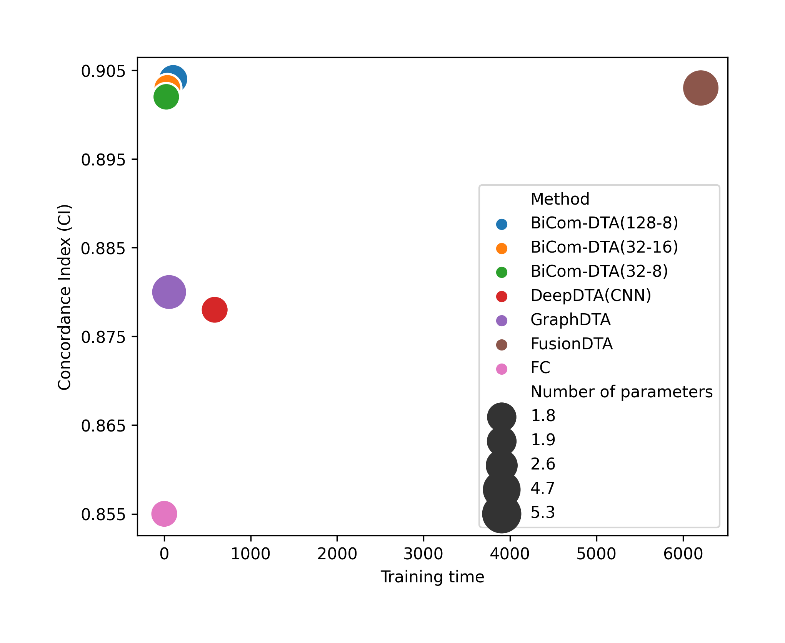


**Fig C in S1 Text. Accuracy vs. training time on CPUs, and the number of parameters for BiComp-DTA and alternative methods**. (A) Training time for Davis dataset, and (B) Training time for Kiba dataset


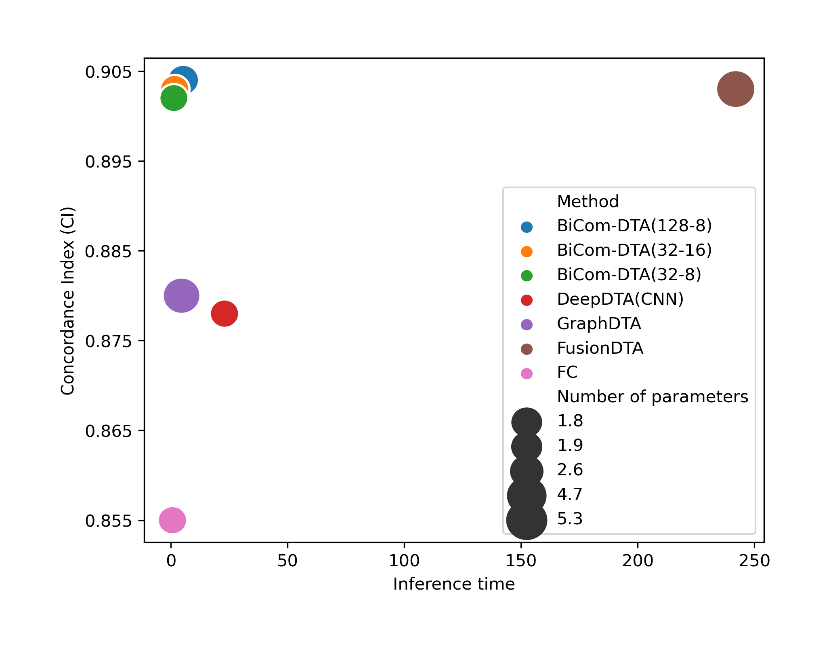

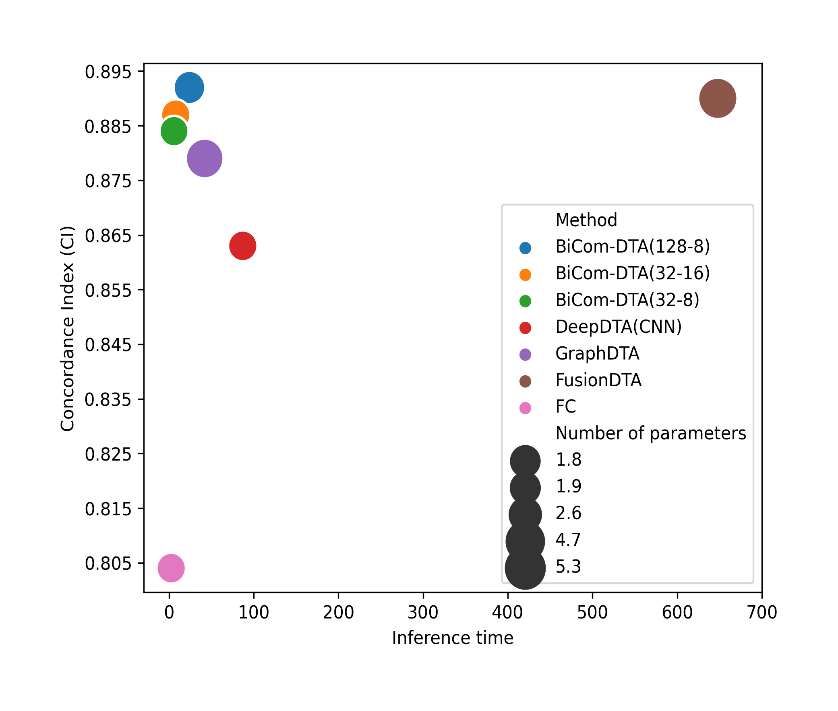


**(A)**

**(B)**

**Fig D in S1 Text. Accuracy vs. inference time on CPUs, and the number of parameters for BiComp-DTA and alternative methods**. (A) Inference time for Davis dataset, and (B) Inference time for Kiba dataset

# References

1. Gönen M, Heller G. Concordance probability and discriminatory power in proportional hazards regression. Biometrika. 2005 Dec 1;92(4):965-70.
2. Roy PP, Roy K. On some aspects of variable selection for partial least squares regression models. QSAR & Combinatorial Science. 2008 Mar;27(3):302-13.
3. Roy K, Chakraborty P, Mitra I, Ojha PK, Kar S, Das RN. Some case studies on application of “rm2” metrics for judging quality of quantitative structure–activity relationship predictions: emphasis on scaling of response data. Journal of computational chemistry. 2013 May 5;34(12):1071-82.
4. Pratim Roy P, Paul S, Mitra I, Roy K. On two novel parameters for validation of predictive QSAR models. Molecules. 2009 May;14(5):1660-701.
5. He T, Heidemeyer M, Ban F, Cherkasov A, Ester M. SimBoost: a read-across approach for predicting drug–target binding affinities using gradient boosting machines. Journal of cheminformatics. 2017 Dec;9(1):1-4.
6. Öztürk H, Özgür A, Ozkirimli E. DeepDTA: deep drug–target binding affinity prediction. Bioinformatics. 2018 Sep 1;34(17):i821-9.
7. Zhao Q, Xiao F, Yang M, Li Y, Wang J. AttentionDTA: prediction of drug–target binding affinity using attention model. In2019 IEEE International Conference on Bioinformatics and Biomedicine (BIBM) 2019 Nov 18 (pp. 64-69). IEEE.
8. Chollet, F, et al. Keras. https://github.com/fchollet/keras. Accessed 18 April 2022.
9. Abadi M, Barham P, Chen J, Chen Z, Davis A, Dean J, Devin M, Ghemawat S, Irving G, Isard M, Kudlur M. Tensorflow: A system for large-scale machine learning. In12th {USENIX} symposium on operating systems design and implementation ({OSDI} 16) 2016 (pp. 265-283).
10. Srivastava N, Hinton G, Krizhevsky A, Sutskever I, Salakhutdinov R. Dropout: a simple way to prevent neural networks from overfitting. The journal of machine learning research. 2014 Jan 1;15(1):1929-58.

1. [↑](#footnote-ref-1)
2. [↑](#footnote-ref-2)
3. [↑](#footnote-ref-3)
4. [↑](#footnote-ref-4)
